# Boric acid as a precatalyst for BH_3_-catalyzed hydroboration†

**DOI:** 10.1039/d1ra05945a

**Published:** 2021-09-28

**Authors:** Julien Légaré Lavergne, Hoang-Minh To, Frédéric-Georges Fontaine

**Affiliations:** Département de Chimie, Université Laval 1045 Avenue de la Médecine Québec G1V 0A6 Québec Canada frederic.fontaine@chm.ulaval.ca

## Abstract

We report that boric acid, BO_3_H_3_, is a good precatalyst for the BH_3_-catalyzed hydroboration of esters using pinacolborane as a borylation agent. Using microwave irradiation as an energy source, we demonstrated that a dozen esters were converted into the corresponding boronate ethers in good yields. It was also possible to use boric acid as a precatalyst to reduce carbonates and alkynes. Considering the hazardous and pyrophoric nature of BH_3_ solutions, boric acid proves to be a safe and green precatalyst for the metal-free reduction of unsaturated species.

## Abstract 2

Cheap and air-stable boric acid is shown to be a good precatalyst for BH_3_ hydroboration of esters and carbonates.

## Graphical abstract

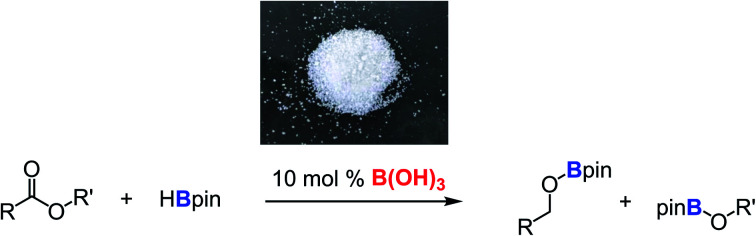

## Introduction

Whereas the reduction of ketones and aldehydes is an important reaction used to generate primary and secondary alcohols, the reduction of esters is a more challenging transformation to achieve.^1,2^ Indeed, mild reductants like NaBH_4_ can easily reduce the former substrates, but the reduction of esters usually requires aggressive reagents such as LiAlH_4_ or BH_3_. Although highly active, these reagents are pyrophoric and come with significant handling risks.^3^ In order to make this operation safer, which is one of the 12 principles of green chemistry, there is interest in developing catalytic systems that use less hazardous chemicals.^4^ Many catalytic systems have been developed for the hydrogenation of esters using first row metal catalysts,^5,6^ but these transformations usually require a high pressure of hydrogen, which is still a substantial safety concern. In parallel to these studies, there has been a surge in the hydrosilylation and hydroboration of esters, which use reagents that are safer to operate on large scale.^7–11^ Although the nature of the reagents differ, there are many similarities in the reactivity of hydroboranes and hydrosilanes in hydrofunctionalization transformations.^12^

Among some of the most active catalysts for ester hydroboration, Marks and collaborators reported the activity of La[N(SiMe_3_)_2_]_3_ with TOF exceeding 60 h^−1^ at 25 °C.^13^ Main group species such as magnesium amide complexes have also shown good catalytic activity with esters and carbonates.^14,15^ In recent reports, it was shown that aldehydes, ketones and carboxylic acids could be reduced using pinacolborane under mild and catalyst-free conditions; however, esters are still elusive substrates under these reducing conditions.^16–20^

**Scheme 1:**
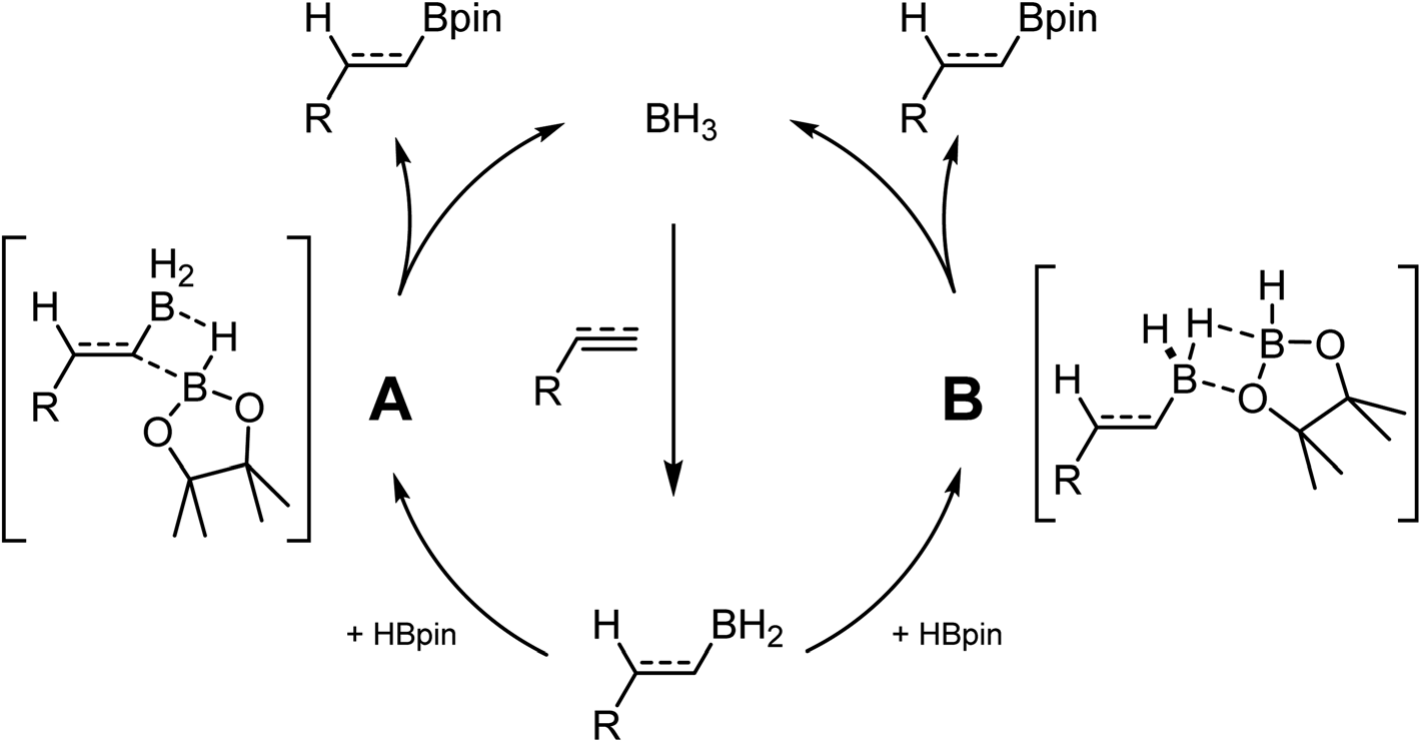
Reaction pathways for the hydroboration of unsaturated substrates.

While investigating the reactivity of frustrated Lewis pair (FLP) aminoborane catalysts for the C–H borylation reaction,^21–23^ we observed that these species could catalyze the hydroboration of electron-poor indoles using HBpin (pin = pinacol).^24^ Further investigation demonstrated that BH_3_ adducts are even more active catalysts for this transformation. In parallel studies, Thomas and coworkers demonstrated that BH_3_ is also an efficient catalyst for the hydroboration of alkenes and alkynes using HBpin.^25^ In another publication they ponder the hidden role of boranes in hydroboration catalysis.^26^ These results are quite impactful, since many hydroboranes are known to undergo rearrangement reactions, notably using transition metal catalysts, generating BH_3_ in the process.^27^ Without proper control studies and high quality reagents, it cannot be excluded that some metal-catalyzed transformations are in fact promoted by the presence of traces of BH_3_ in solution. Based on experimental and computational studies, it was proposed that BH_3_ undergoes first hydroboration of the unsaturated substrate. The R–BH_2_ moiety can then do backbone exchange with HBpin either by cleavage of the B–C bond (Scheme 1 A), or by replacement of the two hydrides with the pinacol (Scheme 1 B).^28–30^

As a logical follow-up on our previous work, we investigated the reactivity of aminoborane FLP catalysts towards the hydroboration of esters. Whereas the activity observed was typical of BH_3_ chemistry, which was already reported to reduce esters,^31^ we were able to demonstrate that boric acid can be used as a precatalyst, generating the active BH_3_ in the process. Boric acid is one of the most benign, abundant and cheapest source of boron and there has been a great interest in the last decade to use it for Lewis acid catalysis.^32^ However, to our knowledge, boric acid has not been used as a precatalyst in reduction chemistry, although it has been recently used as a protonating agent in the metal-free reduction of dinitrogen.^33^ We wish to report in this study our investigation on the reactivity of boric acid as a precatalyst for ester hydroboration.

## Results

**Scheme 2:**
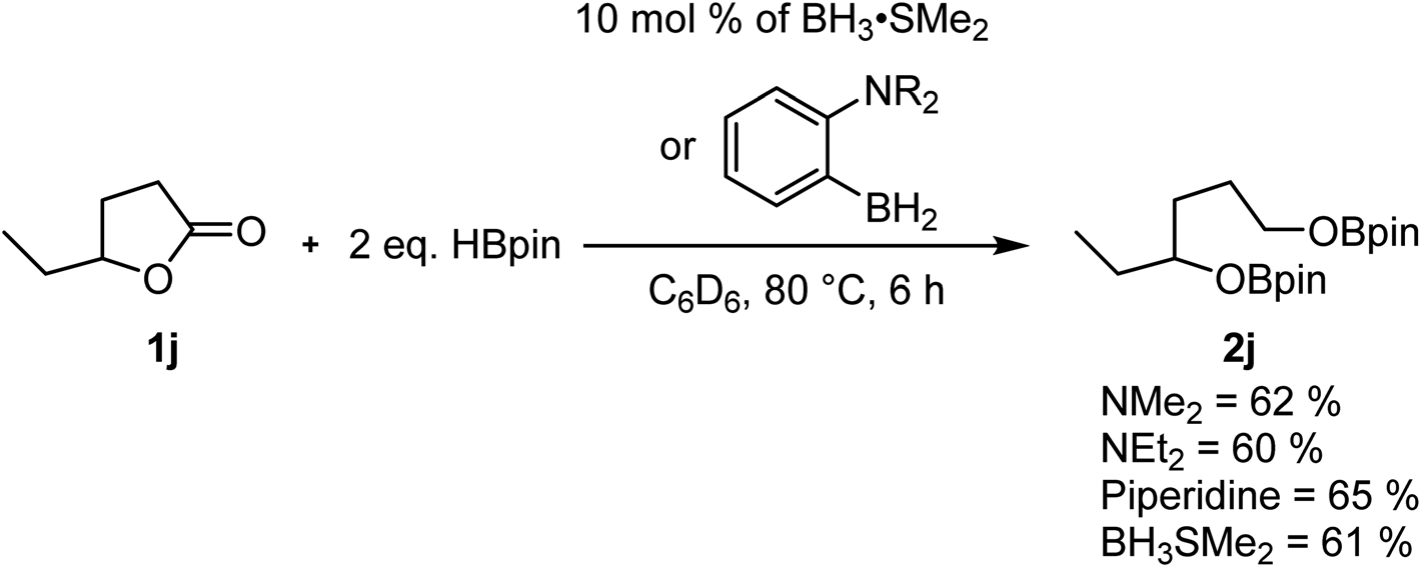
Hydroboration catalysis with aminoboranes and BH_3_•SMe_2_.

Our previous studies on the C–H borylation of heteroarenes using aminoborane catalysts led us to investigate their ability to catalyze the hydroboration of other substrates such as amides and esters.^23^ Initial investigations indicated that the aminoborane species 1-NR_2_-2-BH_2_-C_6_H_4_ (NR_2_ = NMe_2_, NEt_2_ or piperidine) were active catalysts for the hydroboration of γ-caprolactone 1j with HBpin. The size of the amine had little impact on the reaction rate, which led us to believe that the borane moiety was probably the only required functional group for this reaction to operate. Running the experiment using BH_3_·SMe_2_ afforded a comparable yield of the reduction product, suggesting that we were in the presence of a boron reduction process and that the ambiphilic species are not required (Scheme 2). Whereas the BH_3_ adducts are efficient catalysts generating little waste, this class of reagents remains quite precarious to handle and present serious safety concerns. We were able in the past to use –BF_3_ analogues of aminoboranes as a safe alternative to FLP catalysts that could be handled in air, but the preparation of fluoride-protected boranes requires fluoride sources that exhibit some toxicity.^22,23,34,35^ In order to democratize this type of catalytic process, we investigated for safe and cheap alternatives such as boric acid, which under catalytic conditions could generate the desired BH_3_ catalysts.

Boric acid is one of the most convenient and cheapest boron sources, but it has not been widely used under reduction conditions. In order to probe the activity of this reagent as a precatalyst for the hydroboration reaction, we looked at its reactivity in the reaction between benzyl benzoate 1a and HBpin at 200 °C under microwave irradiation to generate BpinOCH_2_(C_6_H_5_) (2a), as shown in Table 1. Without a catalyst in either THF or 2-Me-THF, some of the desired product can be observed. Whereas the yield is only 8% with 2 equiv. of HBpin in 1 h in THF, up to 60% conversion can be observed with 5 equiv. of HBpin for 2 h in 2-Me-THF (Table 1, entries 1–4). The addition of 10 mol% of boric acid significantly increases the yield from 20% to 52% when running the reaction in THF for 1 h with 3 equiv. of HBpin. Under these conditions, increasing the time of the reaction to 2 h only slightly improve the yield (Table 1, entries 5 and 6). With a loading of 20 mol% of boric acid, 85% of the hydroboration product 2a was obtained, while using 5 equiv. of HBpin affords a nearly quantitative yield (Table 1, entries 8 and 9). Other green solvents were tested and proved suitable for this transformation (Table 1, entry 10) but the broad interest of 2-Me-THF by pharmaceutical companies made us continue with that solvent.^36^ However, it should be noted that carbonates should be avoided as a solvent since they can also undergo reduction under catalytic conditions. The temperature seems to be a critical aspect to this reaction, since running the reaction at 150 °C and 100 °C lowered the yield to 76% and 4%, respectively (Table 1, entries 11 and 12). We also monitored the reaction using a 99.999% trace metal boric acid and obtained the same yield, suggesting that the reaction is not promoted by metal impurities (Table 1, entry 13). Boron oxide can also promote this transformation, although a higher loading of precatalyst is required to obtain nearly quantitative yields (Table 1, entries 14 and 15). Interestingly, replacing the boric acid by BH_3_·SMe_2_ did not improve the reaction, suggesting that the rate limiting step is not in the generation of the active borane species (Table 1, entry 16). Therefore, the optimal conditions for testing the scope of the hydroboration of esters were set at 200 °C in 2 mL of 2-Me-THF using 10 mol% of boric acid and 5 equiv. of pinacolborane.

**Table 1:** Catalytic hydroboration of benzyl benzoate using boron precatalysts

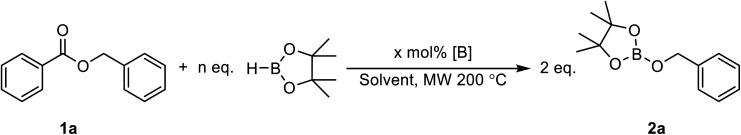						
Entry	Precatalyst	*x* (mol%)	Solvent	*n*	Time (h)	Yield^f^ (%)
1	None	—	THF	2	1	8
2	None	—	THF	3	1	20
3	None	—	2-Me-THF	5	1	35
4	None	—	2-Me-THF	5	2	60
5	BO_3_H_3_	10	THF	3	1	52
6	BO_3_H_3_	10	THF	3	2	63
7	BO_3_H_3_	10	2-Me-THF	3	1	55
8	BO_3_H_3_	20	2-Me-THF	3.2	1	85
9	BO_3_H_3_	10	2-Me-THF	5	1	96
10	BO_3_H_3_	10	CPME^a^	5	1	88
11	BO_3_H_3_	10	2-Me-THF	5	1	4^b^
12	BO_3_H_3_	10	2-Me-THF	5	1	76^c^
13	BO_3_H_3_^d^	10	2-Me-THF	5	1	92
14	B_2_O_3_	10^e^	2-Me-THF	5	1	83
15	B_2_O_3_	20^e^	2-Me-THF	5	1	94
16	BH_3_·SMe_2_	10	2-Me-THF	5	1	92

a Cyclopentyl methyl ether.

b 100 °C.

c 150 °C.

d 99.999% trace metals basis.

e Relative to the amount of boron.

f Yields of benzylOBpin 2a is calculated by integration of product ^1^H NMR signals *versus* mesitylene internal standard.

**Fig. 1:**
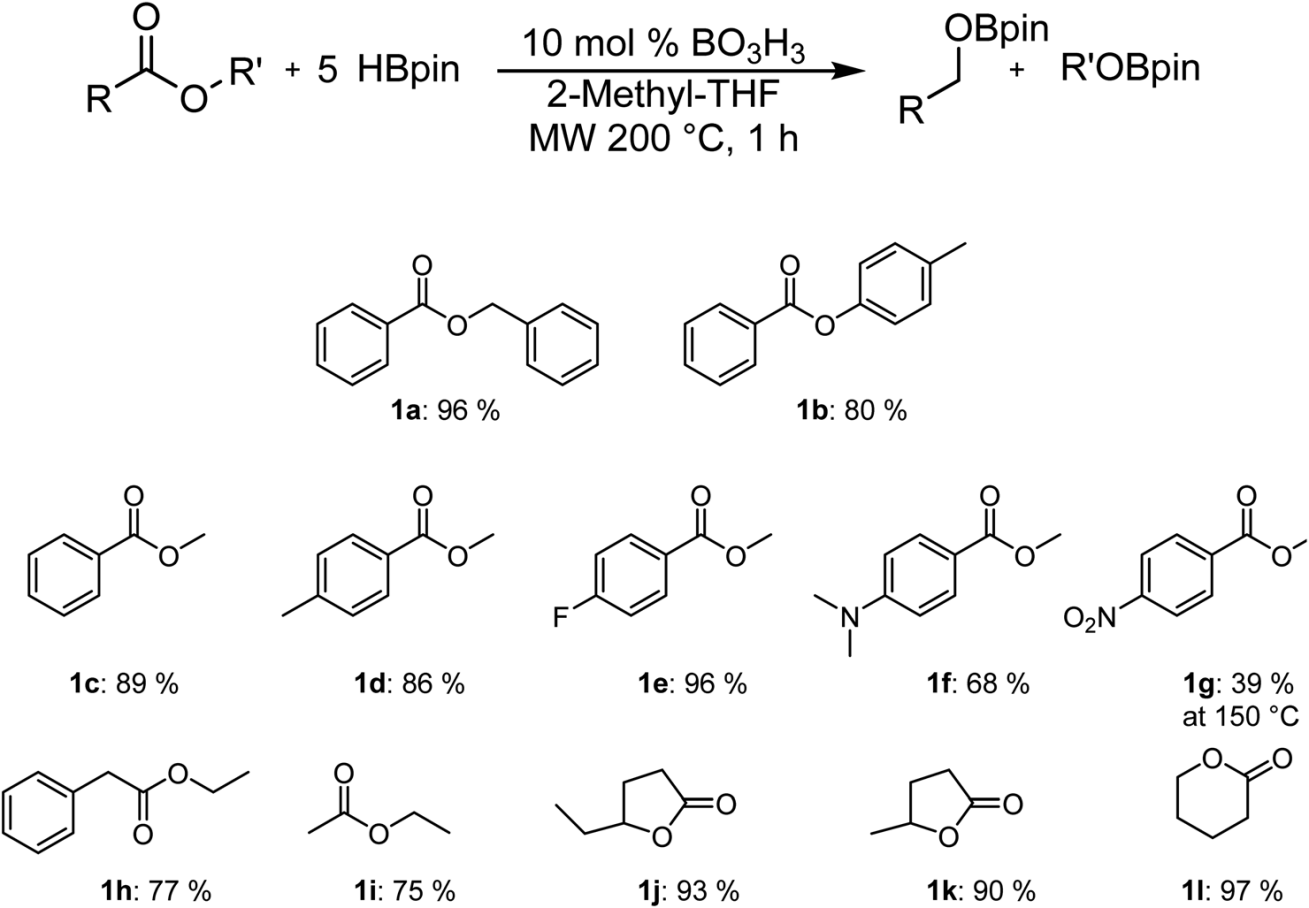
Scope of the hydroboration of esters using boric acid.

As can be seen in Fig. 1, it is possible to reduce several esters in good to excellent yields. The yields were calculated by comparing the integration of the ^1^H NMR resonances to an internal standard (mesitylene). The observed yield of 80% for *p*-tolylbenzoate is only slightly lower than with benzylbenzoate, which was obtained with a 96% yield. The yield for the reduction of the methylbenzanoate derivatives (Ar = phenyl 1c, 4-Me-phenyl 1d, 4-F-phenyl 1e) does not vary significantly from one to the other and range between 86% and 96%. However, the electron-rich 4-NMe_2_-phenyl derivative (1f) is significantly less active than other substrates. It is possible to be selective for the ester over the nitro moiety by carrying the reaction at 150 °C (1g), but the yield of 39% is significantly lower. Good yields were also obtained with ethyl phenylacetate (1h) and ethyl acetate (1i), suggesting that the reaction can also operate with aliphatic moieties instead of aromatic species. Cyclic carboxylic esters are also reduced to their corresponding bisborane products with excellent NMR yields.

**Fig. 2:**
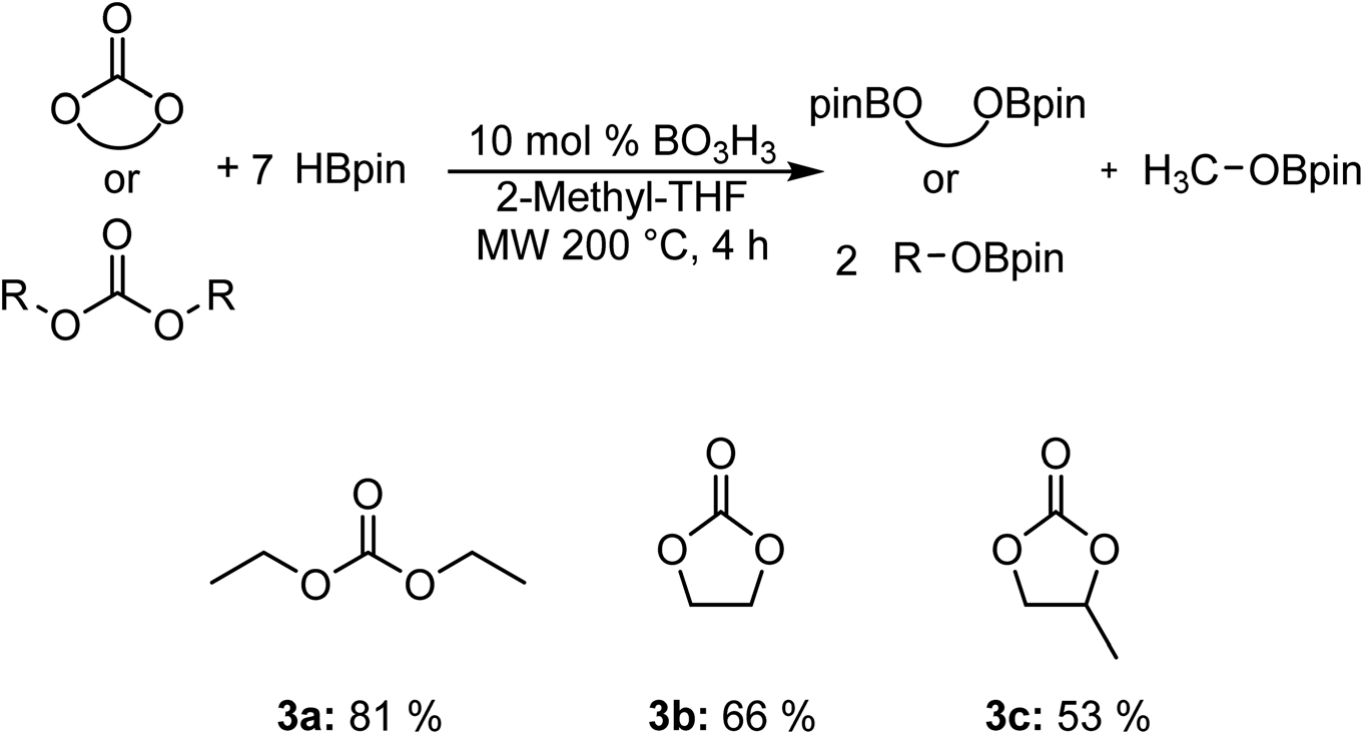
Scope of the hydroboration of carbonates using boric acid.

We also carried out the hydroboration of carbonates using 7 equiv. of HBpin for 4 h to favor the reactivity (Fig. 2). Diethyl carbonate 3a was converted to the corresponding EtOBpin and MeOBpin products with a yield of 81%. The reaction with ethylene carbonate 3b and 1,2-propylene carbonate 3c gave respective yields of 66% and 53%.

## Monitoring of the catalytic transformation

**Fig. 3:**
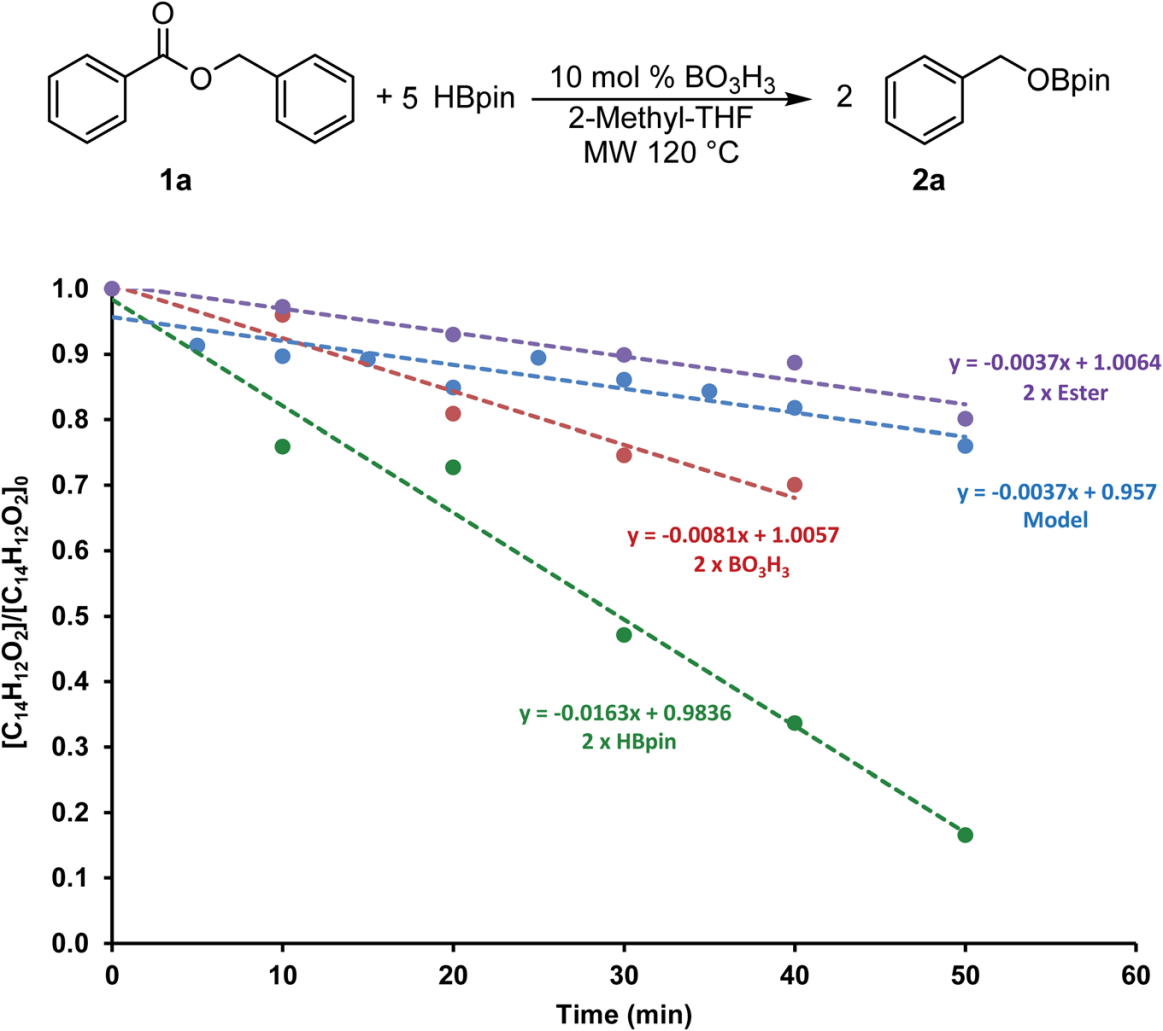
Conversion of the hydroboration of 1a with various concentration of reagents.

To gain more information on this transformation, we looked at the conversion of benzylbenzoate 1a to 2a as a function of time. In a model reaction, 5 equiv. of HBpin was added to 0.60 mmol of 1a at 120 °C in 2 mL of 2-Me-THF using 10 mol% of boric acid (0.06 mmol) as catalyst. Since the monitoring of microwave reactions can be challenging, several experiments were performed by varying the concentration of each of the reagents and stopped using 10 min increments. The yield was then measured by ^1^H NMR spectroscopy using mesitylene as an internal standard. We also monitored this transformation by independently doubling the amount of ester (1.2 mmol), of boric acid (0.12 mmol) and of HBpin (6.0 mmol). The profile of these reactions is shown in Fig. 3.

The consumption of 1a in these four transformations followed a linear trend in the first hour of the transformation. The control experiment showed 20% conversion within the first 50 min of the reaction. The rate of consumption of 1a did not seem to be affected by increasing the quantity of ester added to the system. On the other hand, doubling the concentration of HBpin increased the conversion to almost 85% in the same amount of time, suggesting a reaction four times faster. Although varying the concentration of boric acid in this transformation did affect the reaction rate, it only did by a factor 2. These data suggests that the reaction rate is not dependent on the ester concentration, therefore the hydroboration step is expected to be fast. However, we can presume that the generation of the BH_3_ from the boric acid and/or from the hydroboration product is the most challenging step in this transformation. To get more information on this process, we looked in more details at stoichiometric transformations.

## Stoichiometric studies

**Scheme 3:**
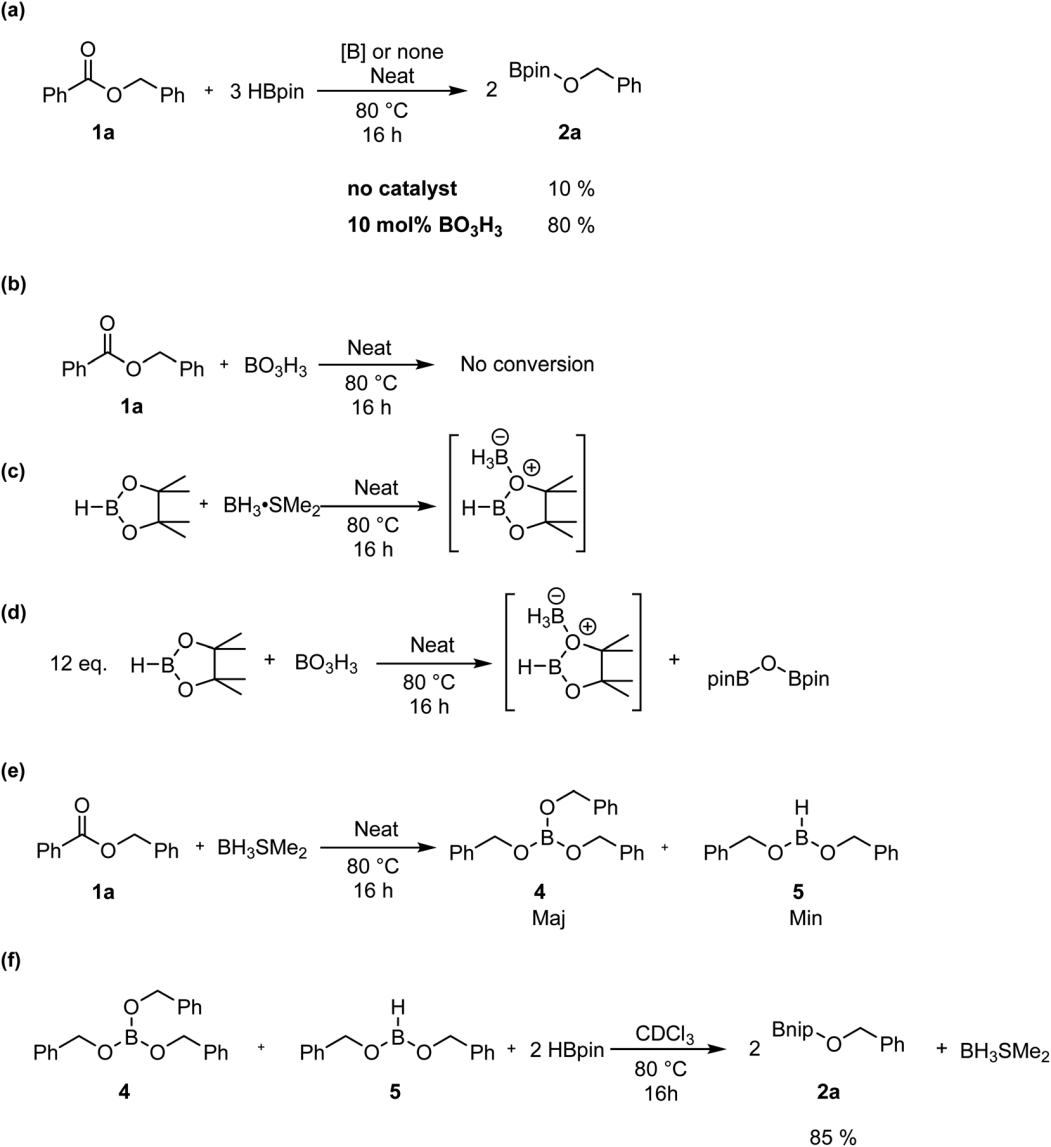
Mechanistic experiments for the hydroboration of esters.

To monitor the role of the boric acid in this transformation, we added 0.3 mmol of benzyl benzoate 1a and 0.9 mmol of HBpin under neat conditions at 80 °C for 16 h in presence of 0.03 mmol of boric acid, then added CDCl_3_ for NMR monitoring (Scheme 3a). As expected, we observed in that period the production of 2a in 80% yield. Running the reaction without any boric acid under the same conditions gave an endemic yield of 10%, supporting the catalytic role of boric acid in this transformation. We also observed by ^1^H NMR spectroscopy the presence of H_2_, assigned as a singlet at 4.6 ppm, when running the reaction with boric acid, which is not the case under catalyst-free conditions. Using ^11^B{^1^H} NMR spectroscopy, benzylOBpin 2a can be observed as a singlet at 22.4 ppm. We were able to observe a resonance at 21.2 ppm, which was attributed to the formation of (Bpin)_2_O,^37^ and a singlet at −13.2 ppm, which was attributed to a BH_3_ adduct. The nature of the latter adduct was supported by the presence of a quartet (*J*^1^_B–H_ = 96 Hz) when carrying out ^11^B NMR, without proton decoupling.^38^ We propose that this species correspond to a BH_3_·HBpin adduct, which is supported by the stoichiometric reaction between BH_3_·SMe_2_ and HBpin, which gives the same multiplet at −13.2 ppm by ^11^B NMR spectroscopy (Scheme 3c). Another reaction was done with boric acid and 12 equiv. of HBpin, which afforded the same adduct in addition to (Bpin)_2_O (Scheme 3d). As expected, we did not observe any reaction between boric acid and benzylbenzoate, which tell us that boric acid is a precatalyst that is only active upon reaction with HBpin (Scheme 3b). These results led us to propose that the active species in this transformation is a borane adduct, which was previously observed in the hydroboration of alkenes, alkynes and indoles.^24^

**Fig. 4:**
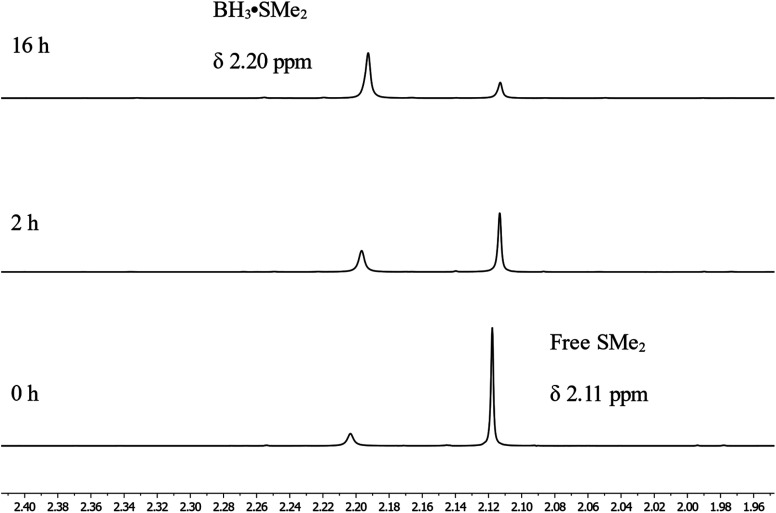
^1^H NMR spectra of the regeneration of BH_3_·SMe_2_ over time.

Looking in more details at the reaction between benzyl benzoate and 1 equiv. of BH_3_·SMe_2_, we can observe the formation of benzyl borate 4 as the major product, as seen in Scheme 3e.^39^ Monitoring by ^11^B NMR spectrum, we can observe a singlet at 18.7 ppm for 4 and a small doublet at 28.2 ppm (*J*^1^_B–H_ = 164 Hz) for what is believed to be the bis(phenylmethyl) borane 5. It is not unusual for RBH_2_ species to undergo rearrangement reactions to generate R_3_B and BH_3_, which could also explain the presence of BH_3_·SMe_2_ under these reaction conditions.^40^ Adding 2 equiv. of HBpin to this reaction led to the consumption of 4 and the production of 2a, as observed by the resonance of the methylene protons shifting from 5.02 ppm to 4.95 ppm (Scheme 3f). A good way to confirm the regeneration of BH_3_ in this transformation is to look at the dimethylsulfide resonance by ^1^H NMR spectroscopy. It can be observed that the concentration of free dimethyl sulfide (*δ* = 2.11) decreases over time to generate the BH_3_·SMe_2_ (*δ* = 2.20), as seen in Fig. 4.

**Scheme 4:**
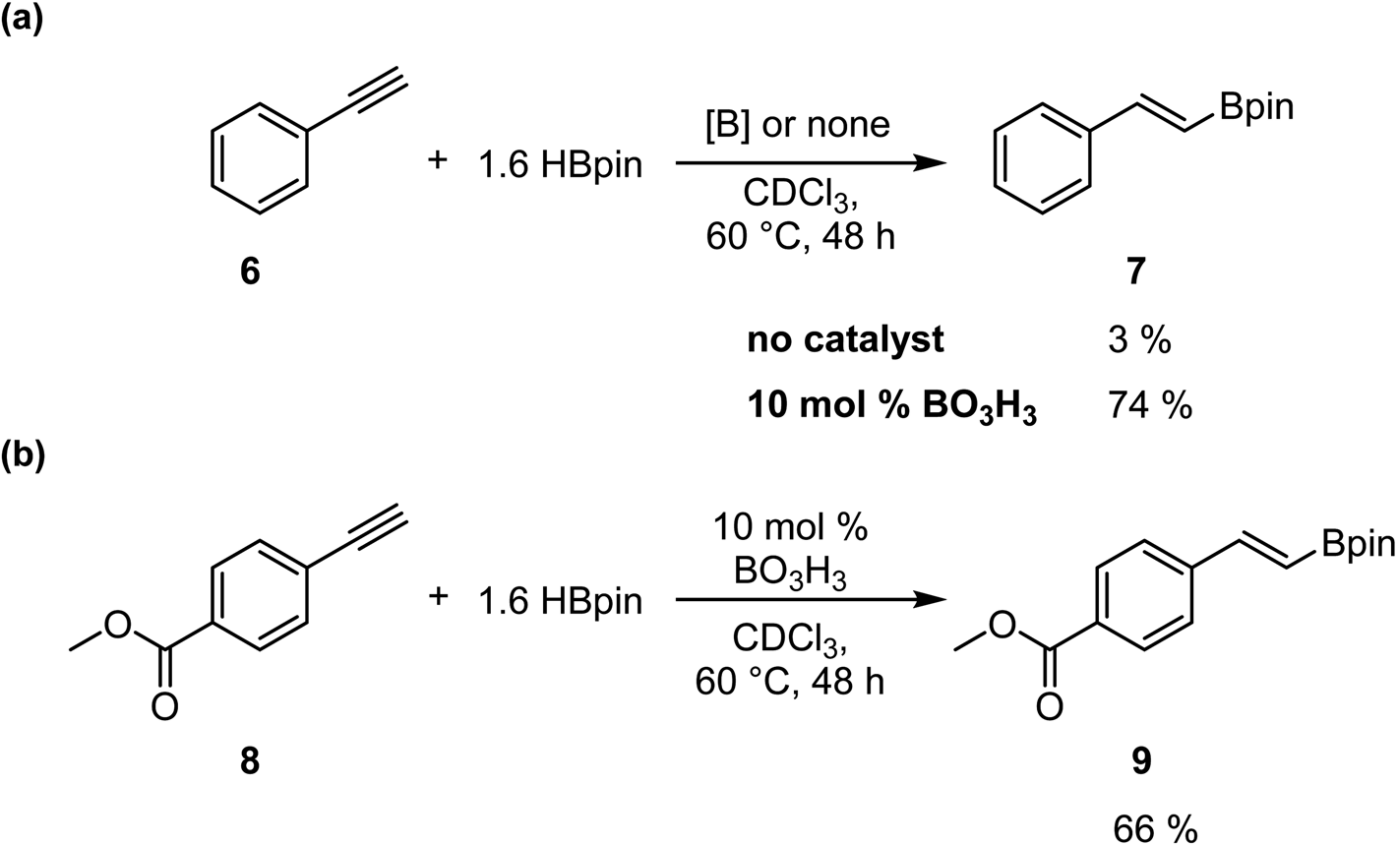
Selective hydroboration of alkynes using boric acid as a precatalyst.

To confirm that this catalytic system allows the generation of BH_3_, which act as the catalyst in this transformation, we investigated the reaction with alkynes, as previously reported by Thomas.^25^ We added in a J-Young NMR tube phenylacetylene 6 with 1.6 equiv. of HBpin and 0.1 equiv. of boric acid in CDCl_3_. We observed the formation of the hydroboration product with an NMR yield of 74%; the same reaction without boric acid only gave 3% conversion (Scheme 4a). However, running the reaction with methyl 4-ethynylbenzoate 8 demonstrate that the hydroboration of the alkyne is preferred over the reduction of the ester moiety, as expected, giving the hydroboration product 9 in 66% yield (Scheme 4b).

## Discussion

**Scheme 5:**
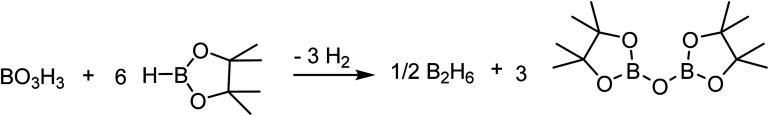
Borane generation from boric acid and pinacolborane.

While the hydroboration of esters by BH_3_ is already described in the literature,^31,40,41^ the experimental evidences support that this step is the fastest in this catalytic system to produce the corresponding alkoxyboranes. The impact of boric acid concentration and of HBpin concentration on the rate of the transformation and the independence of the ester concentration suggest that the rate-limiting step is the generation of the active species from the alkoxyborane and/or boric acid. The activation of the boric acid, shown in Scheme 5, requires 6 equiv. of HBpin, generating H_2_ and (Bpin)_2_O as byproducts. Based on DFT calculations, this conversion from boric acid to BH_3_ is thermodynamically favored by over 50 kcal mol^−1^, but the low reaction rate can be explained by a high-energy barrier and a poor solubility of boric acid in organic solvents.

**Fig. 5:**
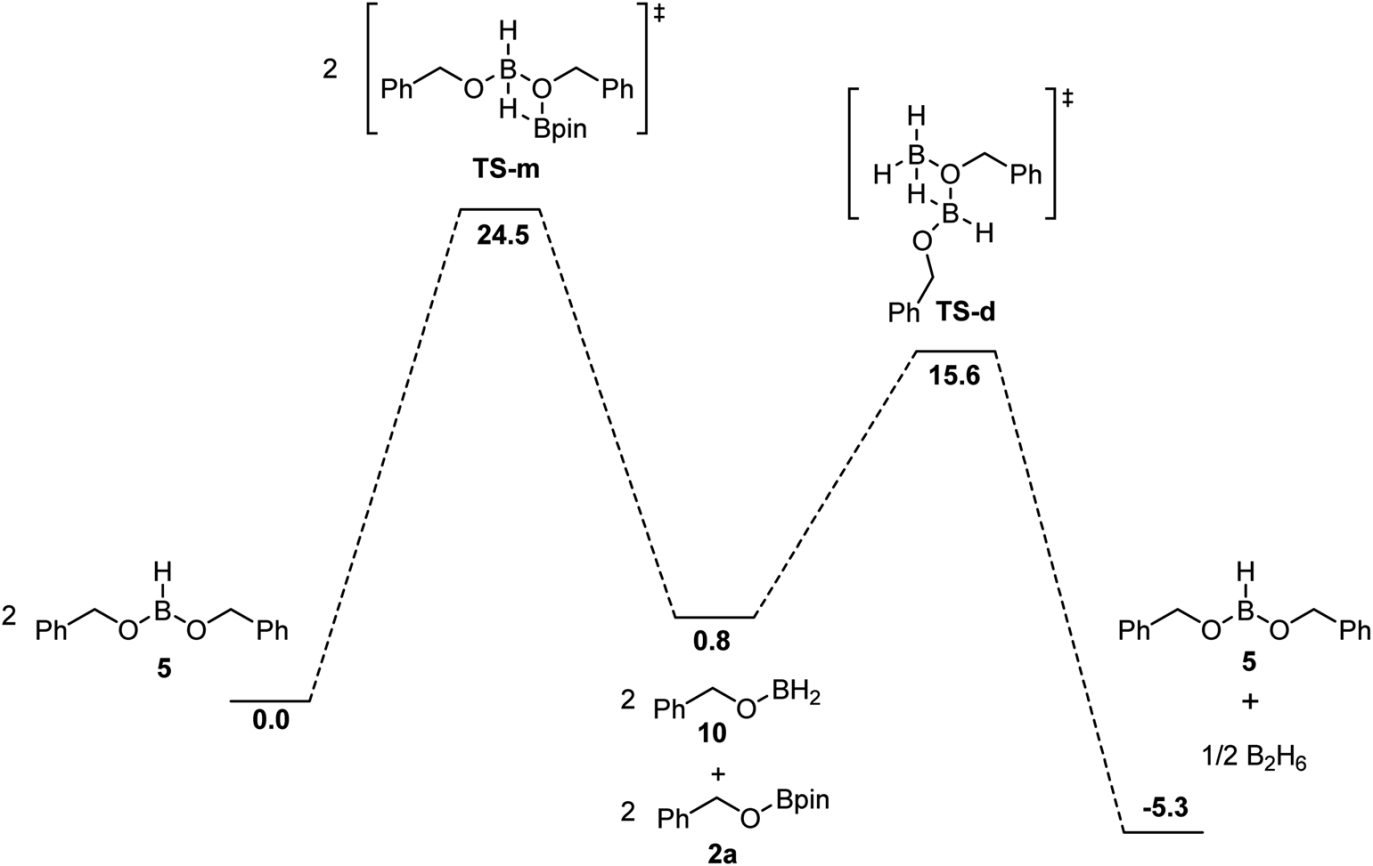
Free energies in kcal mol^−1^ for the proposed mechanism of the formation of 2a and regeneration of BH_3_ calculated at DFT/ωB97XD/6-31+G** (SMD, tetrahydrofuran) level of theory.

BH_3_ can be regenerated by the reaction between HBpin and the dialkoxyborane intermediate. Using DFT calculations, we probed the mechanism of the formation of benzylOBpin 2a and the regeneration of BH_3_, as seen in Fig. 5. A transition step of 24.5 kcal mol^−1^ is proposed for the σ bond metathesis across the B–O bond of the dialkoxyborane 5 and the B–H bond of pinacolborane to generate 2a and benzylOBH_2_. Once 10 is generated, the rearrangement is rapid to generate 5 and BH_3_ with a low barrier of 14.8 kcal mol^−1^.^40^ The overall process is favored thermodynamically by 5.3 kcal mol^−1^. Since the first metathesis is rate limiting, the concentration of HBpin will have an important role on the reaction rate of this transformation.

## Conclusion

Although BH_3_ is well known to hydroborate a large array of reagents, there is a surge of interest in using it as a catalyst for reduction chemistry. However, there are important safety concerns when using BH_3_ adducts since these are highly reactive and pyrophoric molecules, which prevents the scale up of these transformations on very large scale. The ability of using a cheap and highly stable precatalyst like boric acid for reduction chemistry offers a green avenue to BH_3_-catalyzed processes. Using such catalytic process in presence of mild reductant HBpin under microwave conditions, the hydroboration of esters is rapid and lead to good yields, and does not require working under strict air-free conditions. We believe this process to be very user friendly and we are currently investigating to use this system in other reduction processes.

## Conflicts of interest

The authors declare no competing financial interests.

## Supplementary Material

RA-011-D1RA05945A-s001
