# Supplementary material for: Boric acid as a precatalyst for BH3-catalyzed hydroboration
Source: RSC Adv. 2021 Sep 28;11(51):31941–9. doi: 10.1039/d1ra05945a (PMC9041564; doi:10.1039/d1ra05945a)
Supplement: RA-011-D1RA05945A-s001 [file RA-011-D1RA05945A-s001.pdf]

Electronic Supplementary Material (ESI) for RSC Advances.
This journal is © The Royal Society of Chemistry 2021

Supporting Information

Boric acid as a precatalyst for BH_3-catalyzed hydroboration

Julien Légaré Lavergne,[a] Hoang-Minh To,[a] and Frédéric-Georges Fontaine[a]*

a) Département de Chimie, Université Laval, 1045 Avenue de la Médecine, Québec (Québec), Canada, G1V 0A6

Contents

Contents 1
General experimental 1
Initial hydroboration tests with aminoboranes 2
Procedure for the catalytic hydroboration of esters 2
NMR yield calculation 3
Characterization of ester hydroboration products 3
Procedure for the catalytic hydroboration of carbonates 9
Characterization of carbonates hydroboration products 9
Monitoring of the catalytic transformation 10
Stoichiometric studies 11
Procedure for the catalytic hydroboration of alkynes 14
Computational details 15
Cartesian coordinates 15
References 21

General experimental

The synthesis of aminoboranes derivatives 1-NR_2-2-BH_2-C_6H_4 (NR_2 = NMe_2, NEt_2 or piperidine) were done according to reported procedures[1,2] using standard Schlenk techniques. NMR spectra were recorded on Agilent Technologies NMR spectrometer at 500.00 MHz (^{1}H), 125.757 MHz

(^{13}C), 160.46 MHz (^{11}B) and 470.385 MHz (^{19}F) or on Varian Inova NMR AS400 spectrometer, at 400.0 MHz (^{1}H), 100.580 MHz (^{13}C) and 376.29 (^{19}F). ^{1}H NMR and $^{13}C\{^{1}H\}$ NMR chemical shifts are referenced respectively to the residual hydrogen and carbon atoms in the deuterated solvents. $^{11}B\{^{1}H\}$ NMR calibration was performed using $F_3B{\bullet}OEt_2$ as an external reference. ^{19}F NMR was calibrated using $CFCl_3$ as external standard. Multiplicities are reported as singlet (s), broad singlet (s, br) doublet (d), triplet (t), multiplet (m). Chemical shifts are reported in ppm. Coupling constants are reported in Hz. Deuterated-chloroform ($CDCl_3$) was dried by distillation over P_2O_5. Mass spectrometry analyses were carried out on an Agilent 6210 LC Time of Flight Mass Spectrometer, using an electrospray ionization (ESI) method. Esters, carbonates, alkynes and all other chemicals bought from Sigma-Aldrich and were used without further purification. All solvents and boron reagents were used directly from the bottle, without additional purification steps. Microwave reactions were performed with a Monowave 400 from Anton Paar.

Initial hydroboration tests with aminoboranes

Reactions were performed in a J-Young NMR tube using 0.3 mmol of γ-caprolactone **1j** and 0.6 mmol of HBpin under neat conditions at 80 °C for 16 h. To each of these reaction was added 10 mol % of either **1-piperidine-2-BH$_2$-C$_6$H$_4$**,[1] **1-NEt$_2$-2-BH$_2$-C$_6$H$_4$**,[1] **1-NEt$_2$-2-BH$_2$-C$_6$H$_4$**[2] or $BH_3{\bullet}SMe_2$. The yield was determined by adding $CDCl_3$ to the mixture and performing ^{1}H NMR analysis using mesitylene as an internal standard.

Procedure for the catalytic hydroboration of esters

The ester (0.60 mmol, 1 equiv) and 435 µL (3.00 mmol, 5 equiv) of HBpin were dissolved in 2-methyltetrahydrofuran to give a total volume of 2.00 mL, then a catalytic amount of boric acid (3.71 mg, 0.06 mmol, 0.1 equiv) was introduced. All catalytic reactions were carried out using sealable microwave vials. The reaction mixture was subsequently heated at 200 ^{o}C for 1 h. Afterward, all volatiles were evaporated in a rotatory evaporator at 40 ^{o}C. The products and the internal standard were dissolved in $CDCl_3$ and added to a cap-sealed NMR tube for characterization. To measure the NMR yield, 10 µl (0.072 mmol) of mesitylene was weighted and incorporated to the NMR solution.

NMR yield calculation

This is an example on the determination of the NMR yield from a ^{1}H NMR (**Figure S1**) corresponding to **entry 12 of Table 1**. The hydroboration reaction was performed at 150 °C with **1a**.

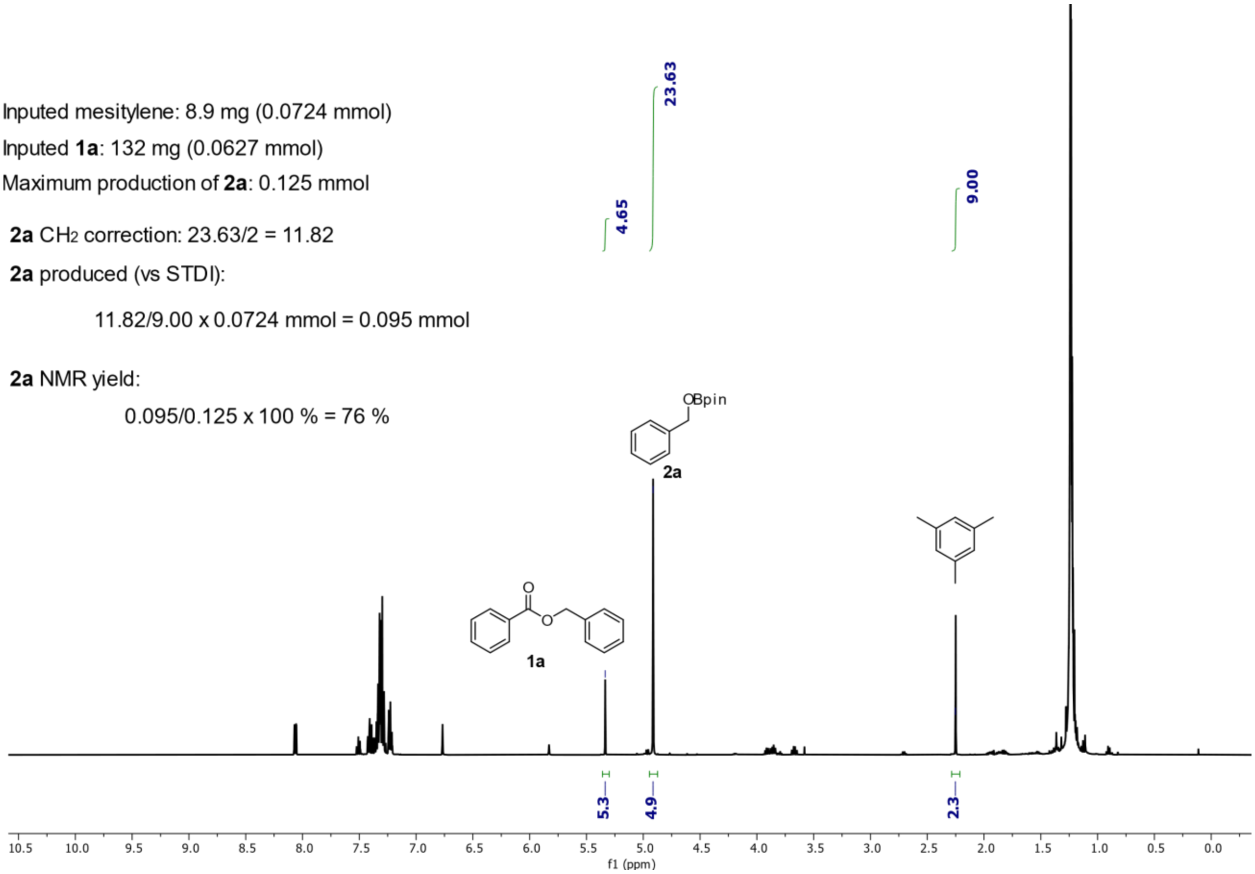

Figure S1 NMR analysis of **entry 12 of Table 1** after the hydroboration catalysis at 150 °C.

Characterization of ester hydroboration products

Characterization data for the products of ester reduction are given below. Reported products were characterized by ^{1}H, ^{11}B{^{1}H}, and ^{13}C{^{1}H} NMR and correspond to the values previously reported for these species.[3,4]

1a Benzyl benzoate

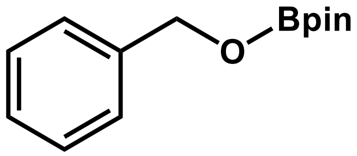

^{1}H NMR (500 MHz, CDCl$_3$): δ 7.29-7.27 (m, 5H, *Ph*CH$_2$OBpin), 4.88 (s, 2H, Ph*CH$_2$*OBpin), 1.23 (s, 12H, PhCH$_2$OB*pin*).

^{11}B{^{1}H} NMR (128 MHz, CDCl$_3$): δ 22.3 (PhCH$_2$O*B*pin).

^{13}C{^{1}H} NMR (125 MHz, CDCl$_3$): δ 139.2 (*Ph*CH$_2$OBpin), 128.0 (*Ph*CH$_2$OBpin), 126.7 (*Ph*CH$_2$OBpin), 126.4 (*Ph*CH$_2$OBpin), 82.7 (PhCH$_2$OB*pin*), 66.5 (Ph*C*H$_2$OBpin), 24.7 (PhCH$_2$OB*pin*).

1b 4-Methyl phenyl benzoate

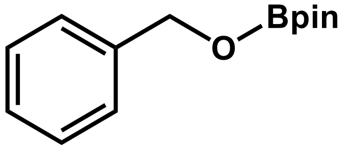

^{1}H NMR (500 MHz, CDCl$_3$): δ 7.29-7.27 (m, 5H, *Ph*CH$_2$OBpin), 4.88 (s, 2H, Ph*CH$_2$*OBpin), 1.21 (s, 12H, PhCH$_2$OB*pin*).

^{11}B{^{1}H} NMR (128 MHz, CDCl$_3$): δ 21.1 (PhCH$_2$O*B*pin).

^{13}C{^{1}H} NMR (125 MHz, CDCl$_3$): δ 139.2 (*Ph*CH$_2$OBpin), 128.2 (*Ph*CH$_2$OBpin), 127.3 (*Ph*CH$_2$OBpin), 126.6 (*Ph*CH$_2$OBpin), 82.9 (PhCH$_2$OB*pin*), 66.5 (Ph*C*H$_2$OBpin), 24.5 (PhCH$_2$OB*pin*).

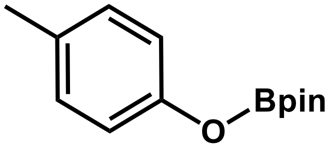

^{1}H NMR (500 MHz, CDCl$_3$): δ 7.01 (d, J = 7.9 Hz, 2H, Me*Ph*OBpin), 6.93 (d, J = 7.9 Hz, 2H, Me*Ph*OBpin), 3.69 (s, 3H, *Me*PhOBpin), 1.26 (s, 12H, MePhOB*pin*).

^{11}B{^{1}H} NMR (128 MHz, CDCl$_3$): δ 21.1 (MePhO*B*pin).

^{13}C{^{1}H} NMR (125 MHz, CDCl$_3$): δ 151.2 (Me*Ph*OBpin), 132.1 (Me*Ph*OBpin), 129.6 (Me*Ph*OBpin), 119.1 (Me*Ph*OBpin), 83.2 (MePhOB*pin*), 55.0 (*Me*PhOBpin), 24.5 (MePhOB*pin*).

1c Methyl benzoate

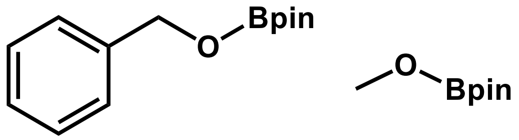

^{1}H NMR (500 MHz, CDCl$_3$): δ 7.29-7.28 (m, 5H, *Ph*CH$_2$OBpin), 4.87 (s, 2H, Ph*CH$_2$*OBpin), 3.55 (s, 3H, *Me*OBpin), 1.21 (s, 12H, PhCH$_2$OB*pin*), 1.20 (s, 12H, MeOB*pin*).

^{11}B{^{1}H} NMR (128 MHz, CDCl$_3$): δ 22.2 (PhCH$_2$O*B*pin/MeO*B*pin).

^{13}C{^{1}H} NMR (125 MHz, CDCl$_3$): δ 128.1 (*Ph*CH$_2$OBpin), 127.2 (*Ph*CH$_2$OBpin), 126.8 (*Ph*CH$_2$OBpin), 126.6 (*Ph*CH$_2$OBpin), 82.9 (PhCH$_2$OB*pin*), 82.7 (MeOB*pin*), 66.5 (Ph*C*H$_2$OBpin), 52.4 (*Me*OBpin), 24.5 (PhCH$_2$OB*pin*), 24.4 (MeOB*pin*).

1d Methyl 4-methyl benzoate

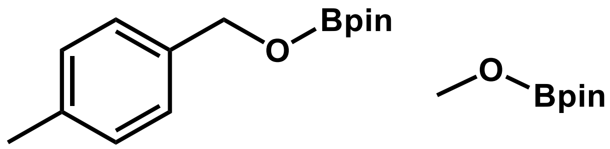

^{1}H NMR (500 MHz, CDCl$_3$): δ 7.20-7.18 (m, 2H, Me*Ph*CH$_2$OBpin), 7.10-7.08 (m, 2H, Me*Ph*CH$_2$OBpin), 4.84 (s, 2H, MePh*CH$_2$*OBpin), 3.56 (s, 3H, *Me*OBpin), 2.29 (s, 3H, *Me*PhCH$_2$Bpin), 1.23 (s, 12H, MePhCH$_2$OB*pin*), 1.22 (s, 12H, MeOB*pin*).

^{11}B{^{1}H} NMR (128 MHz, CDCl$_3$): δ 22.2 (MePhCH$_2$O*B*pin/MeO*B*pin).

^{13}C{^{1}H} NMR (125 MHz, CDCl$_3$): δ 136.7 (Me*Ph*CH$_2$OBpin), 136.2 (Me*Ph*CH$_2$OBpin), 128.8 (Me*Ph*CH$_2$OBpin), 126.7 (Me*Ph*CH$_2$OBpin), 82.8 (MePhCH$_2$OB*pin*), 82.6 (MeOB*pin*), 66.4 (MePh*C*H$_2$OBpin), 52.3 (*Me*OBpin), 24.4 (MePhCH$_2$OB*pin*), 24.4 (MeOB*pin*), 20.9 (*Me*PhCH$_2$OBpin).

1e Methyl 4-fluoro benzoate

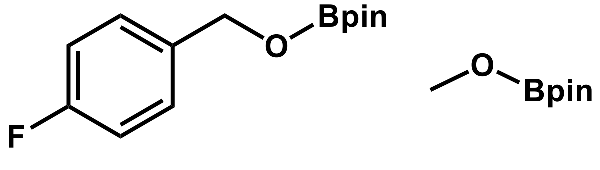

^{1}H NMR (500 MHz, $CDCl_3$): δ 7.15-7.12 (m, 2H, F*Ph*CH$_2$OBpin), 6.84-6.80 (m, 2H, F*Ph*CH$_2$OBpin), 4.68 (s, 2H, FPh*CH$_2$*OBpin), 3.40 (s, 3H, *Me*OBpin), 1.07 (s, 12H, FPhCH$_2$OB*pin*), 1.06 (s, 12H, MeOB*pin*).

^{11}B{^{1}H} NMR (128 MHz, $CDCl_3$): δ 22.1 (FPhCH$_2$O*B*pin/MeO*B*pin).

^{13}C{^{1}H} NMR (125 MHz, $CDCl_3$): δ 162.9 (F*Ph*CH$_2$OBpin), 160.9 (F*Ph*CH$_2$OBpin), 128.4 (F*Ph*CH$_2$OBpin), 114.9 (d, F*Ph*CH$_2$OBpin), 82.7 (FPhCH$_2$OB*pin*), 82.3 (MeOB*pin*), 65.7 (FPh*C*H$_2$OBpin), 52.1 (*Me*OBpin), 24.3 (MeOB*pin*), 24.2 (FPhCH$_2$OB*pin*).

1f Methyl 4-(dimethylamino) benzoate

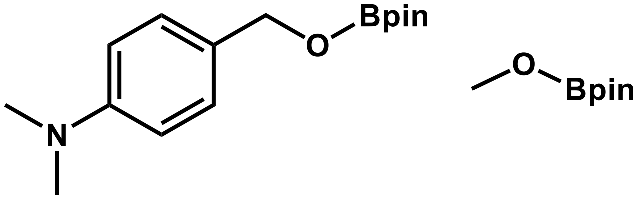

^{1}H NMR (500 MHz, $CDCl_3$): δ 6.97-6.95 (m, 2H, Me$_2$N*Ph*CH$_2$OBpin), 6.60-6.59 (m, 2H, Me$_2$N*Ph*CH$_2$OBpin), 3.53 (s, 3H, *Me*OBpin), 2.82 (s, 6H, *Me$_2$*NPhCH$_2$Bpin), 1.19 (s, 12H, Me$_2$NPhCH$_2$OB*pin*), 1.18 (s, 12H, MeOB*pin*).

^{11}B{^{1}H} NMR (128 MHz, $CDCl_3$): δ 21.1 (Me$_2$NPhCH$_2$O*B*pin/MeO*B*pin).

^{13}C{^{1}H} NMR (125 MHz, $CDCl_3$): δ 148.7 (Me$_2$N*Ph*CH$_2$OBpin), 129.4 (Me$_2$N*Ph*CH$_2$OBpin), 126.8 (Me$_2$N*Ph*CH$_2$OBpin), 113.1 (Me$_2$N*Ph*CH$_2$OBpin), 82.9 (Me$_2$NPhCH$_2$OB*pin*/MeOB*pin*), 67.2 (Me$_2$NPh*C*H$_2$OBpin), 52.3 (*Me*OBpin), 40.8 (*Me$_2$*NPhCH$_2$OB*pin*), 24.5 (Me$_2$NPhCH$_2$OB*pin*), 24.4 (MeOB*pin*).

1g Methyl 4-nitro benzoate

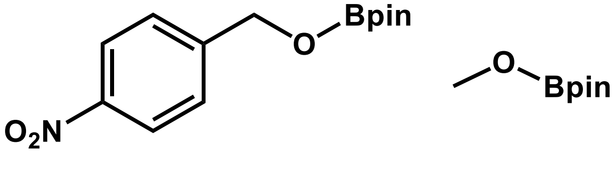

^{1}H NMR (500 MHz, $CDCl_3$): δ 8.17-8.05 (m, 2H, NO$_2$*Ph*CH$_2$OBpin), 7.40-7.39 (m, 2H, NO$_2$*Ph*CH$_2$OBpin), 4.90 (s, 2H, NO$_2$Ph*CH$_2$*OBpin), 3.45 (s, 3H, *Me*OBpin), 1.13 (s, 12H, NO$_2$PhCH$_2$OB*pin*), 1.12 (s, 12H, MeOB*pin*).

^{11}B{^{1}H} NMR (128 MHz, CDCl$_3$): δ 21.0 (NO$_2$PhCH$_2$O*B*pin/MeO*B*pin).

^{13}C{^{1}H} NMR (125 MHz, CDCl$_3$): δ 146.5 (NO$_2$*Ph*CH$_2$OBpin), 130.5 (NO$_2$*Ph*CH$_2$OBpin), 126.7 (NO$_2$*Ph*CH$_2$OBpin), 123.3 (NO$_2$*Ph*CH$_2$OBpin), 83.8 (NO$_2$PhCH$_2$OB*pin*), 82.5 (MeOB*pin*), 65.3 (NO$_2$Ph*C*H$_2$OBpin), 52.5 (*Me*OBpin), 24.7 (MeOB*pin*), 24.3 (NO$_2$PhCH$_2$OB*pin*).

1h Ethyl 2-phenylacetate

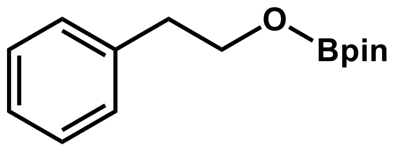

^{1}H NMR (500 MHz, CDCl$_3$): δ 7.14-7.02 (m, 5H, *Ph*CH$_2$CH$_2$OBpin), 4.02 (t, *J* = 6.9 Hz, 2H, PhC*H$_2$*CH$_2$OBpin), 3.90-3.85 (m, 2H, PhCH$_2$C*H$_2$*OBpin), 1.11 (s, 12H, PhCH$_2$CH$_2$OB*pin*).

^{11}B{^{1}H} NMR (128 MHz, CDCl$_3$): δ 22.0 (PhCH$_2$CH$_2$O*B*pin).

^{13}C{^{1}H} NMR (125 MHz, CDCl$_3$): δ 138.3 (*Ph*CH$_2$CH$_2$OBpin), 128.9 (*Ph*CH$_2$CH$_2$OBpin), 128.1 (*Ph*CH$_2$CH$_2$OBpin), 126.0 (*Ph*CH$_2$CH$_2$OBpin), 82.3 (PhCH$_2$CH$_2$OB*pin*), 65.4 (Ph*C*H$_2$CH$_2$OBpin), 37.0 (PhCH$_2$*C*H$_2$OBpin) 24.3 (PhCH$_2$CH$_2$OB*pin*).

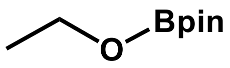

^{1}H NMR (500 MHz, CDCl$_3$): δ 3.91 (q, *J* = 7.0 Hz, 2H, CH$_3$C*H$_2$*OBpin), 1.12 (s, 12H, EtOB*pin*) 1.08 (t, *J* = 7.0 Hz, 3H, *CH$_3$*CH$_2$OBpin).

^{11}B{^{1}H} NMR (128 MHz, CDCl$_3$): δ 22.0 (EtO*B*pin).

^{13}C{^{1}H} NMR (125 MHz, CDCl$_3$): δ 82.7 (EtOB*pin*), 60.3 (CH$_3$*C*H$_2$OBpin), 24.4 (EtOB*pin*), 17.0 (*C*H$_3$CH$_2$OBpin).

1i Ethyl acetate

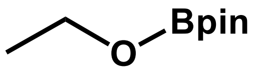

^{1}H NMR (500 MHz, CDCl$_3$): δ 3.90 (q, *J* = 7.0 Hz 2H, CH$_3$*CH$_2$*OBpin), 1.19 (s, 12H, CH$_3$CH$_2$OB*pin*), 1.12 (t, *J* = 7.0 Hz, 3H, *CH$_3$*CH$_2$OBpin).

^{11}**B{^{1}H} NMR (128 MHz, CDCl$_3$):** δ 22.0 (CH_3CH_2O*B*pin).

^{13}**C{^{1}H} NMR (125 MHz, CDCl$_3$):** δ 82.4 (CH_3CH_2OB*pin*), 60.5 (CH_3*C*H_2OBpin), 24.5 (CH_3CH_2OB*pin*), 17.1 (*C*H_3CH_2OBpin).

1j γ-Caprolactone

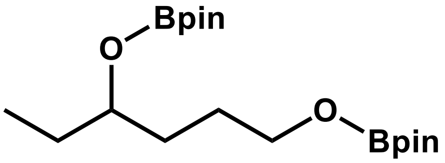

^{1}**H NMR (500 MHz, CDCl$_3$):** δ 3.92-3.84 (m, 1H), 3.79-3.74 (m, 2H,), 1.60-1.29 (m, 6H), 1.19-1.17 (s, 24H), 0.83 (t, *J* = 7.3 Hz, 3H).

^{11}**B{^{1}H} NMR (128 MHz, CDCl$_3$):** δ 21.1 (CH_3CH_2CH(O*B*pin)$(CH_2)_3$O*B*pin).

^{13}**C{^{1}H} NMR (125 MHz, CDCl$_3$):** δ 82.5, 82.3, 75, 64.8, 31.8, 29.2, 27.4, 24.4, 9.6.

1k γ-Valerolactone

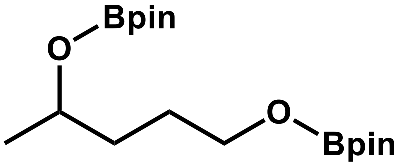

^{1}**H NMR (500 MHz, CDCl$_3$):** δ 4.12-4.07 (m, 1H), 3.77-3.73 (m, 2H), 1.46-1.43 (m, 2H), 1.18-1.16 (s, 24H), 1.10 (d, *J* = 6.1 Hz, 2H), 0.83-0.79 (m, 3H).

^{11}**B{^{1}H} NMR (128 MHz, CDCl$_3$):** δ 21.0 (CH_3CH(O*B*pin)$(CH_2)_3$O*B*pin).

^{13}**C{^{1}H} NMR (125 MHz, CDCl$_3$):** δ 82.5, 82.3, 70.4, 64.6, 34.0, 27.4, 24.5, 24.4, 22.4.

1l δ-Valerolactone

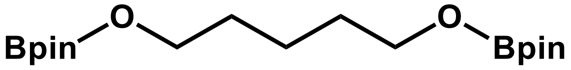

^{1}**H NMR (500 MHz, CDCl$_3$):** δ 3.88-3.59 (m, 6H, BpinO(*C*$H_2)_5$OBpin), 1.54-1.46 (m, 4H, BpinO(*C*$H_2)_5$OBpin), 1.18-1.16 (s, 24H, B*pin*O$(CH_2)_5$OB*pin*).

^{11}**B{^{1}H} NMR (128 MHz, CDCl$_3$):** δ 22.0 (*B*pinO$(CH_2)_5$O*B*pin).

$^{13}C\{^1H\}$ NMR (125 MHz, $CDCl_3$): δ 82.5 (BO*C*Me$_2$), 64.7 (*C*H$_2$OBpin), 31.0 (*C*H$_2$CH$_2$OBpin), 24.5 (BOC*Me*$_2$), 21.6 (*C*H$_2$CH$_2$CH$_2$OBpin).

Procedure for the catalytic hydroboration of carbonates

Carbonate substrates (0.60 mmol, 1 equiv) and 609 µL (4.2 mmol, 7 equiv) of HBpin were dissolved in 2-methyltetrahydrofuran to give a total volume of 2.00 mL, then a catalytic amount of boric acid (3.71 mg, 0.06 mmol, 0.1 equiv) was introduced. All catalytic reactions were carried out using sealable microwave vials. The reaction mixture was subsequently heated at 200 ^{o}C for 4 h. Afterward, all volatiles were evaporated in a rotatory evaporator at 40 ^{o}C. The products and the internal standard (mesitylene) were dissolved in $CDCl_3$ and added to a cap-sealed NMR tube, wrapped with parafilm for NMR characterization.

Characterization of carbonates hydroboration products

Characterization data for the products of carbonates reduction are given below. Reported products were characterized by ^1H, $^{11}B\{^1H\}$, and $^{13}C\{^1H\}$ NMR.[5]

3a Ethylene carbonate

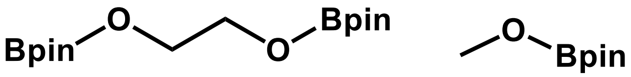

^1H NMR (500 MHz, $CDCl_3$): δ 3.86 (s, 4H, BpinO*CH*$_2$*CH*$_2$OBpin), 3.52 (s, 3H, *Me*OBpin), 1.19-1.18 (m, 36H, B*pin*O(CH$_2$)$_2$OB*pin* and MeOB*pin*).

$^{11}B\{^1H\}$ NMR (128 MHz, $CDCl_3$): δ 22.2 (*B*pinO(CH$_2$)$_2$O*B*pin/MeO*B*pin).

$^{13}C\{^1H\}$ NMR (125 MHz, $CDCl_3$): δ 82.6 (B*pin*OCH$_2$CH$_2$OB*pin*), 82.5 (MeOB*pin*), 67.5 (BpinOCH$_2$*C*H$_2$OBpin), 64.9 (BpinO*C*H$_2$CH$_2$OBpin), 52.5 (*Me*OBpin), 24.7 (MeOB*pin*), 20.8 (B*pin*OCH$_2$CH$_2$OB*pin*).

3b Diethyl carbonate

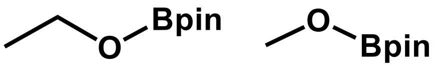

^{1}H NMR (500 MHz, $CDCl_3$): δ 3.89 (q, *J* = 7.1 Hz, 2H, $CH_3\mathit{CH_2}$OBpin), 3.59 (s, 3H, $\mathit{CH_3}$OBpin), 1.25 (s, 12H, MeOB*pin*), 1.24 (s, 12H, CH_3CH_2OB*pin*), 1.20 (t, *J* = 7.1 Hz, 3H, $\mathit{CH_3}CH_2$OBpin).

^{11}B{^{1}H} NMR (128 MHz, $CDCl_3$): δ 22.0 (CH_3CH_2O*B*pin/MeO*B*pin).

^{13}C{^{1}H} NMR (125 MHz, $CDCl_3$): δ 83.1 (MeOB*pin*), 82.5 (CH_3CH_2OB*pin*), 60.6 ($CH_3\mathit{C}H_2$OBpin), 52.6 (*Me*OBpin), 25.0 (MeOB*pin*), 24.5 (CH_3CH_2OB*pin*), 17.1 ($\mathit{C}H_3CH_2$OBpin).

3c Propylene carbonate

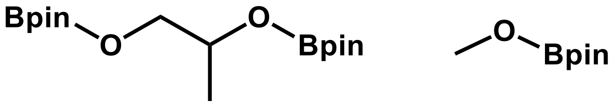

^{1}H NMR (500 MHz, $CDCl_3$): δ 4.18-4.14 (m, 1H), 3.85-3.74 (m, 2H), 3.48 (s, 3H, *Me*OBpin), 1.15-1.13 (m, 36H, B*pin*O$CH_2CH(CH_3)$OB*pin* and MeOB*pin*), 1.06 (d, *J* = 6.0 Hz, 3H).

^{11}B{^{1}H} NMR (128 MHz, $CDCl_3$): δ 21.0 (*B*pinO$CH_2CH(CH_3)$O*B*pin and MOe*B*pin).

^{13}C{^{1}H} NMR (125 MHz, $CDCl_3$): δ 82.8, 82.4 (MeOB*pin*), 67.5, 52.4 (*Me*OBpin), 32.9, 24.4 (B*pin*O$CH_2CH(CH_3)$OB*pin* and MeOB*pin*), 20.8.

Monitoring of the catalytic transformation

A time study of the catalytic transformation was carried out for benzyl benzoate **1a** using boric acid as catalyst by collecting 6 data points early in the reaction. A model reaction was first performed with 132 mg of **1a** (0.60 mmol, 1 equiv), 435 µL (3.0 mmol, 5 equiv) of HBpin and 3.71 mg (0.06 mmol, 0.1 equiv) of boric acid. Three other reactions were done by doubling the amount of one of the reagents: a reaction with 264 mg of **1a** (1.20 mmol, 2 equiv), another with 870 µL (6.00 mmol, 10 equiv) of HBpin and a fourth reaction with 7.42 mg (0.12 mmol, 0.2 equiv) of boric acid. Under these conditions, the reaction can be approximated as a zeroth order with respect to the substrate concentrations. The consumption of **1a** was measured by ^{1}H NMR with the area of the peak (CH_2; 5.37 ppm) relative to a mesitylene internal standard. The reactions were done at 120 ^{o}C using Monowave 400. Orders for HBpin and benzyl benzoate were approximated by doubling the concentration of the corresponding reagent. The order for catalyst was approximated from the rates of reduction at 2 different catalyst loadings (10-20%). The rates (r)

were measured as the slope of the line for consumption of benzylbenzoate ([**1a**]/[**1a**]$_0$) over time (t).

$$\frac{[\mathbf{1a}]}{[\mathbf{1a}]_0} = \mathrm{rt}$$

Stoichiometric studies

Reactions **a-d** described in **Scheme 3** were performed following a model reaction in a J-Young NMR tube using 0.3 mmol of benzyl benzoate **1a**, 0.9 mmol of HBpin under neat conditions at 80 °C for 16 h.

a) Reactions with and without 10 mol% of boric acid afforded NMR yields of 80 % and 10 %. ^{11}B NMR of the catalyzed shows a small quadruplet at -13.2 ppm (J = 96 Hz) as seen in **Figure S2**. The catalyzed reaction performed in a closed J-Young tube in $CDCl_3$ shows a singlet at 4.6 ppm characteristic for molecular hydrogen.

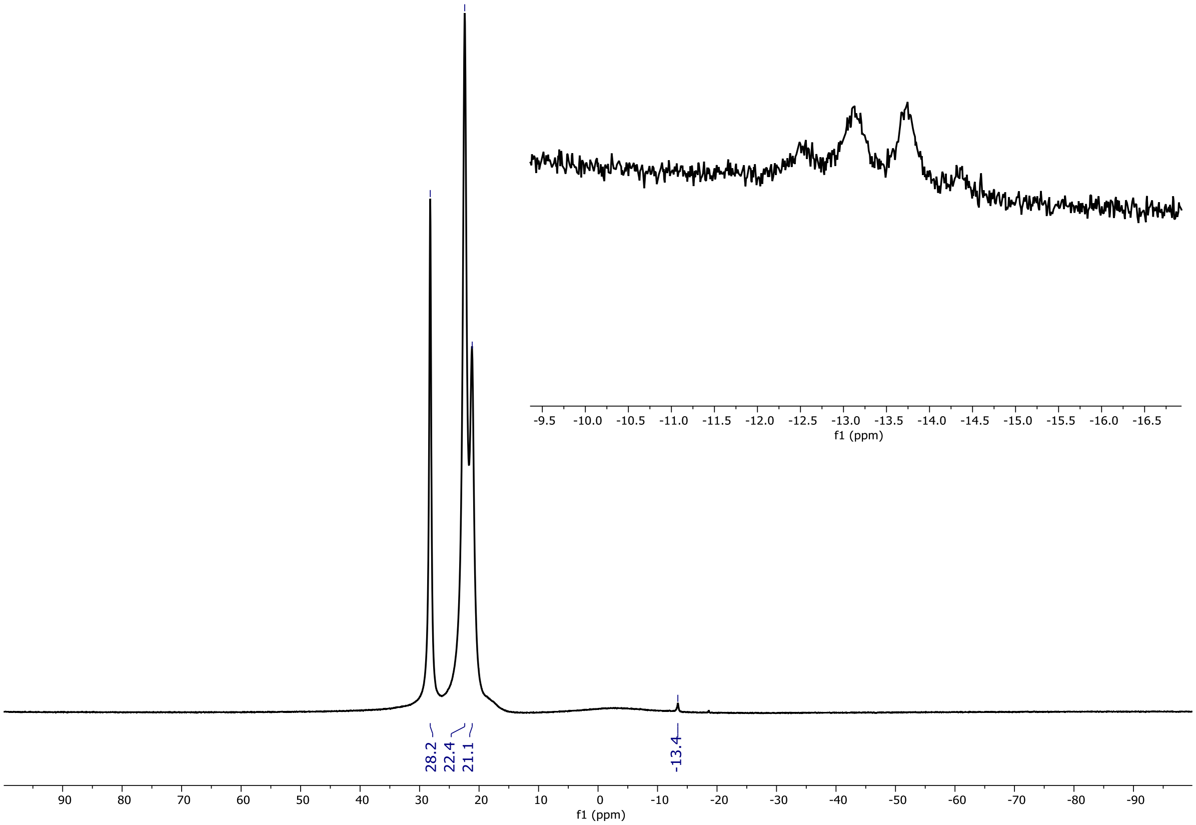

Figure S2. ^{11}B{^{1}H} NMR and ^{11}B NMR (inlet) of the catalyzed reaction shown in **Scheme 3a**.

b) No transformation was observed for the reaction between 1 equiv of benzyl benzoate **1a** and 1 equiv of boric acid under the same conditions than **a)**.

c) The reaction between 1 equiv of HBpin and 1 equiv of BH_3•SMe_2 produces a little amount of multiple small singlets in $^{11}B\{^1H\}$ NMR. In ^{11}B NMR it is possible to see a multiplet at -13.2 ppm corresponding to the speculated adduct (**Figure S3**).

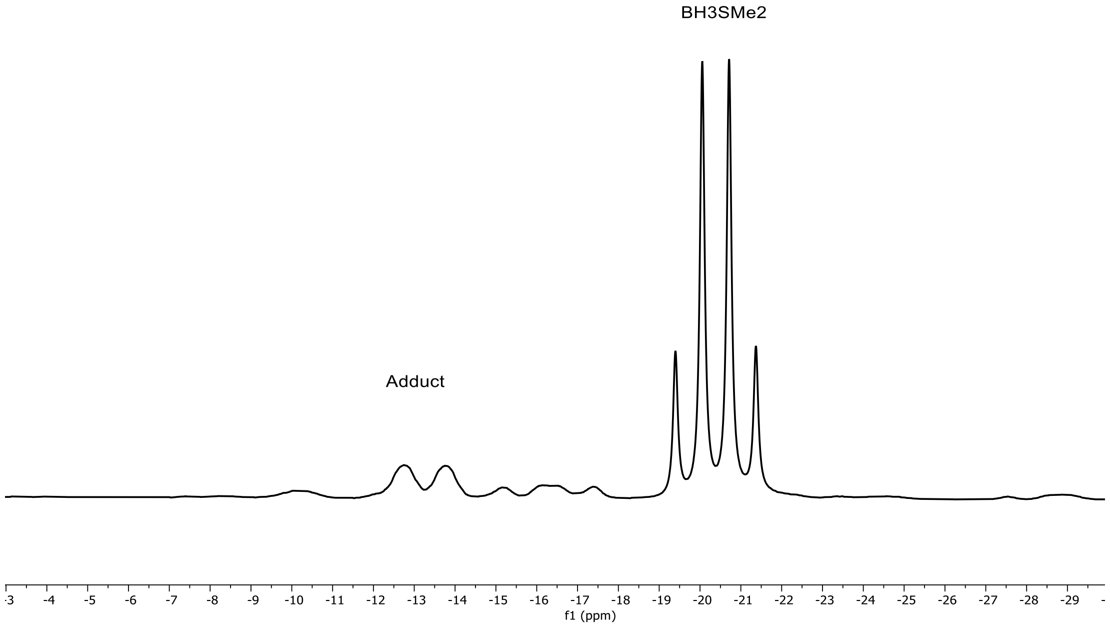

Figure S3. ^{11}B NMR of reaction **c**.

d) The reaction between 12 equiv of HBpin and 1 equiv of BO_3H_3 produces the speculated adduct, $(Bpin)_2O$ and an unidentified compound in ^{11}B NMR (**Figure S4**).

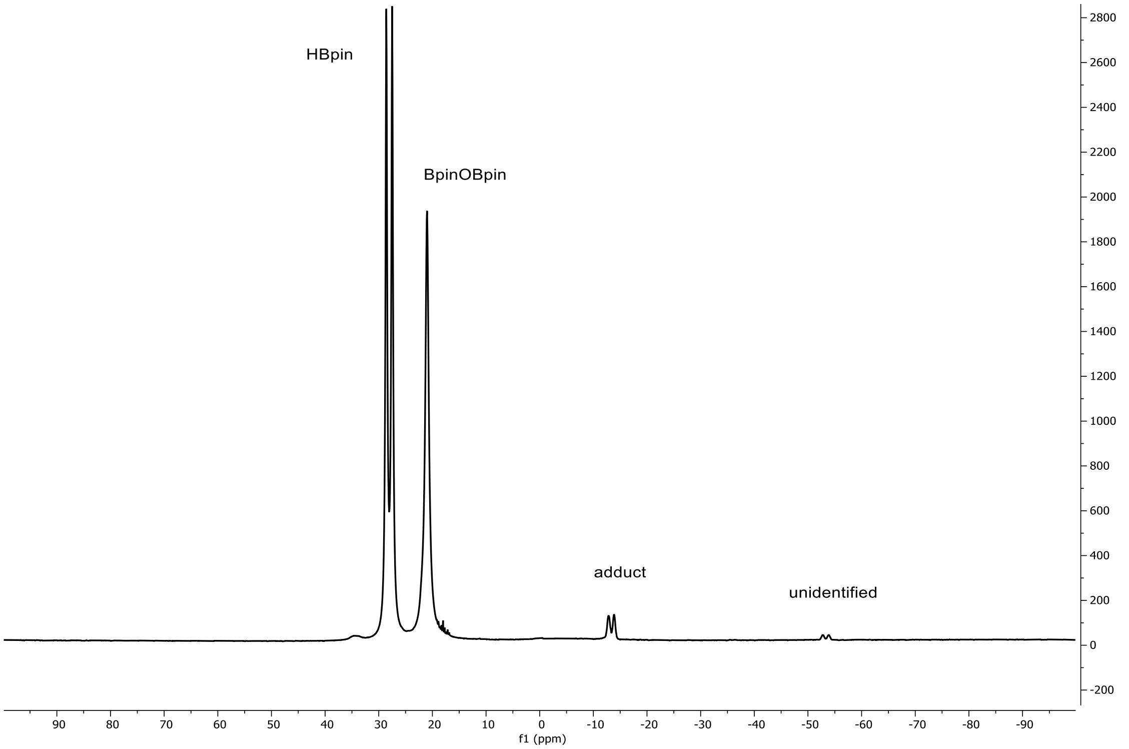

Figure S4 ^{11}B NMR of reaction **d**.

e) The reaction between benzyl benzoate **1a** and 1 equiv of BH_3•SMe_2 was monitored. After 16h, four products are present by ^{11}B NMR: BH_3•SMe_2, benzyl borate **4**, bis(phenylmethyl) boronate **5** and the adduct observed in **c)** at -13.2 ppm (**Figure S5**). Heating the mixture after adding 2 equiv of HBpin produces the benzylOBpin **2a** and regenerates BH_3•SMe_2 overtime.

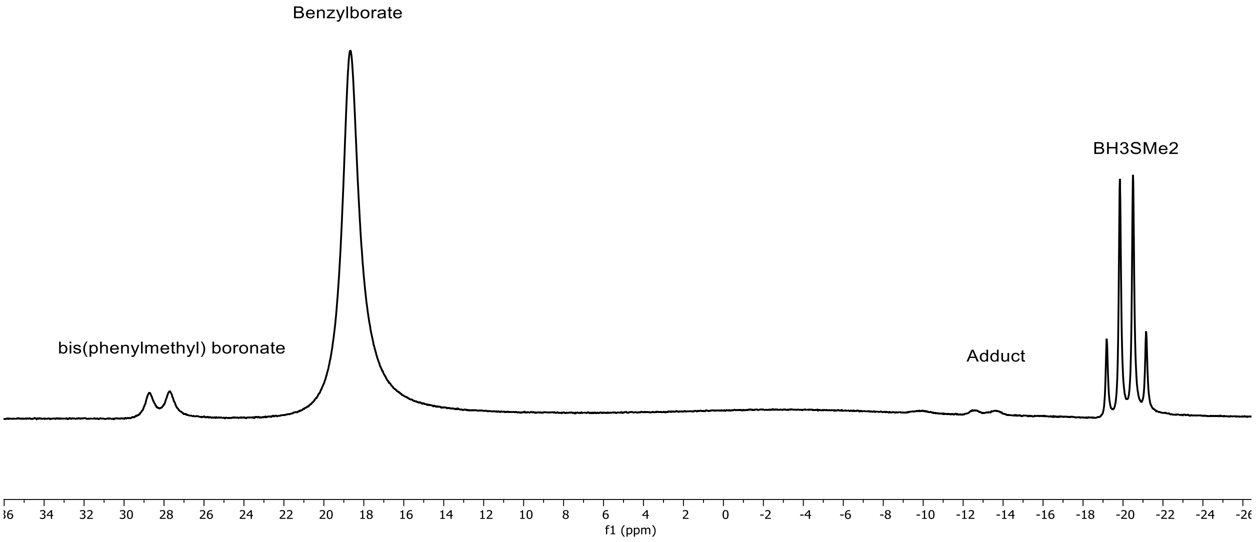

Figure S5. ^{11}B NMR of reaction **d**.

Procedure for the catalytic hydroboration of alkynes

Alkyne substrates (0.32 mmol, 1 equiv) and HBpin (75.0 µL; 0.52 mmol, 1.6 equiv) were dissolved in $CDCl_3$ with a total volume of 0.4 mL in a J-Young tube. A catalytic amount of boric acid (2.0 mg, 0.03 mmol, 0.1 equiv) and 5 µL of mesitylene as internal standard were added to the tube. The reaction mixture was subsequently heated at 60 °C for 48 h. NMR yields were calculated using ^1H NMR spectroscopy.[6]

7 (E)-4,4,5,5-Tetramethyl-2-(phenyl-1-enyl)-1,3,2-dioxaborolane

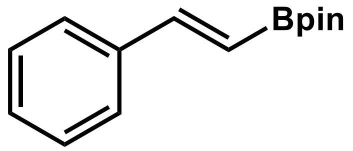

^1H NMR (500 MHz, $CDCl_3$): δ 7.52–7.47 (m, 2 H), 7.41 (d, *J* = 18.4 Hz, 1 H), 7.36–7.27 (m, 3 H), 6.18 (d, *J* = 18.4 Hz, 1 H), 1.32 (s, 12 H).

$^{11}B\{^1H\}$ NMR (128 MHz, $CDCl_3$): δ 29.0.

$^{13}C\{^1H\}$ NMR (125 MHz, $CDCl_3$): δ = 149.6, 137.6, 129.0, 128.7, 127.2, 116.4 (br, C-B), 83.5, 25.0.

9 Methyl 4-[(E)-2-(4,4,5,5-tetramethyl-2-yl)ethenyl]benzoate-1,3,2-dioxaborolane

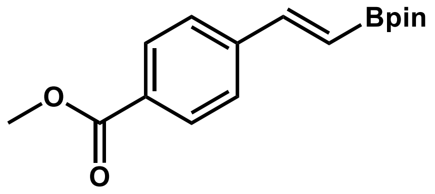

^1H NMR (500 MHz, $CDCl_3$): δ 8.11–7.90 (m, 2 H), 7.55–7.52 (m, 2 H), 7.41 (d, *J* = 18.3 Hz, 1 H), 6.22 (d, J = 18.2 Hz, 1 H), 3.83 (s, 3 H), 1.26 (s, 12 H).

$^{11}B\{^1H\}$ NMR (128 MHz, $CDCl_3$): δ 28.1.

$^{13}C\{^1H\}$ NMR (125 MHz, $CDCl_3$): δ 166.6, 148.0, 141.6, 130.1, 129.8, 126.8, 119.5 (br, C-B), 83.4, 52.0, 24.8.

Computational details

All calculations were performed on the full structures of the reported compounds using the Gaussian 16 suite of programs.[7] The ωB97XD functional[8] was used in combination with the 6-31+G** basis set for all atoms. All geometry optimizations were carried out without any symmetry constraints. The transition states were located and confirmed by frequency calculations (single imaginary frequency). Similarly, the stationary points were characterized as minima (no imaginary frequency). The energies were then refined by single point calculations to include solvent effect using the SMD solvation model[9] with the experimental solvent (tetrahydrofuran) at the same level of theory.[10] All structures with their associated free enthalpy and Gibbs free energies as well as their Cartesian coordinates are fully detailed in the following section.

Cartesian coordinates

BO_3H_3

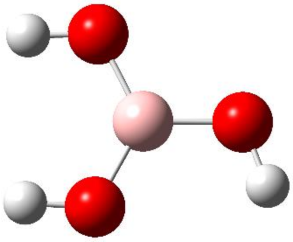

Sum of electronic and thermal Enthalpies= -252.384419

Sum of electronic and thermal Free Energies= -252.416814

B	0.54026	0.15505	-0.02899
O	-0.07106	1.34692	-0.31710
H	-1.02777	1.34871	-0.28556
O	1.90171	0.13840	-0.07011
H	2.25740	-0.72738	0.14242
O	-0.11654	-1.01562	0.29640
H	-1.07340	-0.98062	0.31989

H_2

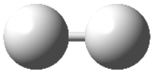

Sum of electronic and thermal Enthalpies= -1.161066

Sum of electronic and thermal Free Energies= -1.175857

H	-0.16970	0.54471	0.02916
H	-0.91220	0.54471	0.02916

HBpin

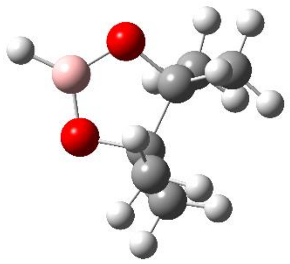

Sum of electronic and thermal Enthalpies= -411.583431

Sum of electronic and thermal Free Energies= -411.626594

C	-0.86461	0.50266	-0.05325	H	-1.17392	-1.48241	-0.85384
C	0.70448	0.50595	0.01952	H	-2.54442	-0.37827	-1.06689
B	-0.08027	2.62859	-0.10750	H	-1.11435	-0.20961	-2.09313
H	-0.08036	3.81612	-0.15807	C	1.37260	0.17267	-1.31552
O	-1.15954	1.86367	-0.44994	H	2.44190	0.38461	-1.23644
O	0.99904	1.89579	0.29881	H	1.24552	-0.88312	-1.57174
C	-1.53290	0.28436	1.30523	H	0.96564	0.78107	-2.12818
H	-2.60219	0.48872	1.20813	C	1.29379	-0.35459	1.12761
H	-1.40589	-0.74570	1.65066	H	1.01435	-1.40382	0.98595
H	-1.12616	0.95997	2.06306	H	2.38446	-0.28511	1.10454
C	-1.45374	-0.44912	-1.08410	H	0.95421	-0.03033	2.11261

(Bpin)$_2$O

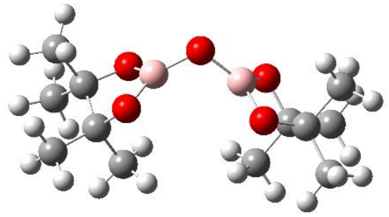

Sum of electronic and thermal Enthalpies= -897.303677

Sum of electronic and thermal Free Energies= -897.370417

O	0.15961	0.28873	0.09593	H	0.08678	5.31564	1.06008
C	-2.31971	2.83424	0.30023	H	-0.56290	4.95509	-0.54427
C	-1.08254	3.51165	0.99505	C	-2.52492	3.29995	-1.14266
C	3.50852	-0.78681	-0.44798	H	-3.26286	2.64979	-1.61947
C	3.31076	0.28559	-1.58079	H	-2.89174	4.32976	-1.18168
O	2.15589	-1.01661	-0.00148	H	-1.59592	3.23815	-1.71705
O	2.11561	0.97004	-1.14890	C	3.00476	-0.33331	-2.94580
O	0.00277	2.64716	0.59759	H	3.88752	-0.82047	-3.36998
O	-1.92170	1.44843	0.24447	H	2.19875	-1.06978	-2.87789
B	-0.55169	1.42905	0.29078	H	2.68452	0.45911	-3.62724
B	1.43669	0.10402	-0.32782	C	4.44045	1.29723	-1.70496
C	-3.62393	2.94279	1.07637	H	5.38311	0.79141	-1.93860
H	-3.90103	3.99320	1.21399	H	4.21808	1.99764	-2.51438
H	-4.42401	2.44788	0.51960	H	4.56619	1.87115	-0.78549
H	-3.54840	2.46699	2.05551	C	4.10473	-2.10567	-0.91726
C	-1.15414	3.46842	2.52246	H	5.09858	-1.94465	-1.34784
H	-0.18518	3.77034	2.92839	H	4.20608	-2.78524	-0.06697
H	-1.91887	4.15033	2.90551	H	3.47146	-2.58869	-1.66324
H	-1.37436	2.45937	2.88291	C	4.29190	-0.25179	0.75244
C	-0.78323	4.92715	0.52405	H	3.88465	0.70198	1.10068
H	-1.63373	5.58610	0.72777	H	4.21560	-0.97256	1.57053

H	5.34927	-0.11208	0.50942

B_2H_6

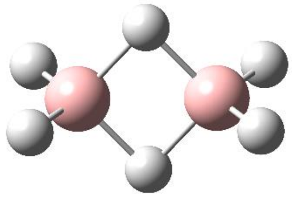

Sum of electronic and thermal Enthalpies= -53.204311

Sum of electronic and thermal Free Energies= -53.231966

B	-2.46886	1.57400	0.18012
H	-1.36492	1.23119	0.81203
H	-2.24595	2.52887	-0.49806
H	-2.98190	0.59420	-0.26490
H	-3.13139	1.95621	1.25289
B	-2.02785	1.61179	1.88493
H	-1.51469	2.59161	2.32988
H	-2.25064	0.65696	2.56311

$HB(OBenzyl)_2$ (5)

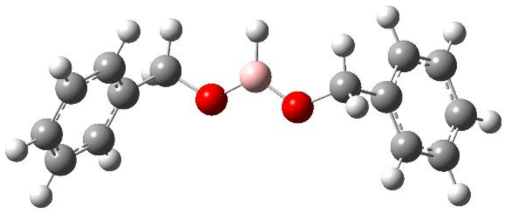

Sum of electronic and thermal Enthalpies= -717.450462

Sum of electronic and thermal Free Energies= -717.501094

B	-0.57650	0.75234	0.57668
H	-1.45257	-0.05582	0.40930
O	-0.80176	1.91616	1.23741
O	0.68162	0.54067	0.11373
C	-2.10461	2.26825	1.66063
H	-1.99585	2.76137	2.63264
H	-2.71795	1.37003	1.80909
C	1.03396	-0.68959	-0.48835
H	0.15075	-1.17071	-0.92827
H	1.72502	-0.45268	-1.30447
C	-2.79539	3.20059	0.68887
C	-2.07905	3.90880	-0.27565
C	-4.17809	3.38092	0.77899
C	-2.73770	4.78445	-1.13791
H	-1.00545	3.77034	-0.34894
C	-4.83528	4.25826	-0.07834
H	-4.74693	2.82707	1.52251
C	-4.11513	4.96342	-1.04179
H	-2.16997	5.32867	-1.88646
H	-5.91047	4.38653	0.00065
H	-4.62635	5.64484	-1.71454
C	1.69848	-1.63976	0.48460
C	2.21884	-1.19343	1.69900
C	1.82102	-2.99004	0.14640
C	2.85186	-2.08633	2.56293
H	2.12526	-0.14569	1.96477
C	2.45673	-3.88064	1.00617
H	1.41162	-3.34958	-0.79506
C	2.97432	-3.43011	2.22003
H	3.25102	-1.72778	3.50682
H	2.54230	-4.92770	0.73223
H	3.46652	-4.12396	2.89424

TS-m

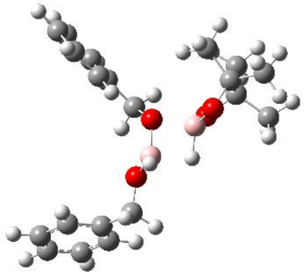

Sum of electronic and thermal Enthalpies= -1129.012615

Sum of electronic and thermal Free Energies= -1129.08859

B	-0.02465	-1.72524	-2.16730	O	0.11733	-1.29355	-0.78334
H	0.58629	-1.11259	-2.99443	O	-0.40703	-3.02061	-2.34647
C	0.15892	-2.28935	0.26599	C	-0.14723	-3.70279	-3.56709
H	-0.68258	-2.97442	0.15265	H	-0.00073	-2.97684	-4.37581
H	-1.40678	-0.80854	-2.22512	H	-1.03462	-4.29848	-3.79810
B	-1.27451	-0.40129	-1.03746	C	1.06575	-4.59166	-3.43404
O	-1.05181	0.97927	-0.95808	C	2.34615	-4.03117	-3.42506
O	-2.23744	-0.81753	-0.09868	C	0.93013	-5.97222	-3.29329
C	-1.65404	1.42838	0.26265	C	3.46961	-4.83730	-3.28327
C	-2.79605	0.37125	0.48009	H	2.46173	-2.95471	-3.52267
C	-2.14508	2.85756	0.06888	C	2.05489	-6.78498	-3.15536
C	-0.58969	1.39943	1.36491	H	-0.06201	-6.41656	-3.29362
C	-3.13254	0.09385	1.93936	C	3.32604	-6.21820	-3.15236
C	-4.07203	0.70973	-0.29588	H	4.45786	-4.38883	-3.26984
H	-2.70845	3.19517	0.94563	H	1.93649	-7.85935	-3.05207
H	-1.28934	3.52501	-0.06593	H	4.20343	-6.84862	-3.04463
H	-2.78136	2.94176	-0.81370	C	1.47631	-3.01503	0.23645
H	0.26487	1.99892	1.04030	C	1.51284	-4.40581	0.14111
H	-0.96984	1.81284	2.30415	C	2.67617	-2.30339	0.31578
H	-0.23373	0.38345	1.55446	C	2.72998	-5.08187	0.14686
H	-3.47280	1.00803	2.43758	H	0.58442	-4.96071	0.04282
H	-3.93527	-0.64651	1.99786	C	3.89415	-2.97654	0.30899
H	-2.26930	-0.29935	2.48026	H	2.65115	-1.21865	0.37489
H	-4.73938	-0.15575	-0.26509	C	3.92209	-4.36843	0.23096
H	-4.59469	1.56752	0.13836	H	2.74599	-6.16326	0.05971
H	-3.84739	0.92943	-1.34352	H	4.82182	-2.41564	0.36917
H	0.02812	-1.73161	1.19466	H	4.87204	-4.89415	0.22482

H_2BOBenzyl (10)

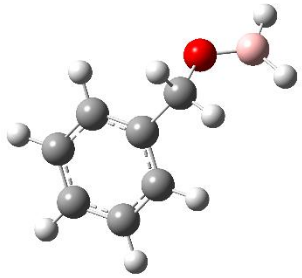

Sum of electronic and thermal Enthalpies= -372.01281

Sum of electronic and thermal Free Energies= -372.053661

B	-2.34294	1.36785	-0.13700	O	-1.43019	0.73149	0.62069
H	-2.49479	2.55786	-0.02740	C	-0.58425	1.43658	1.53402
H	-2.98120	0.69773	-0.89460	H	-1.03840	2.40135	1.78745

H	-0.53031	0.82524	2.43887
C	0.78792	1.63841	0.94486
C	1.73191	0.60949	0.97993
C	1.12075	2.84471	0.32585
C	2.98670	0.78174	0.40272
H	1.47810	-0.33307	1.45752

C	2.37645	3.02167	-0.25122
H	0.39162	3.65048	0.29432
C	3.31114	1.98953	-0.21354
H	3.71280	-0.02454	0.43559
H	2.62501	3.96483	-0.72770
H	4.29063	2.12591	-0.66116

BenzylOBpin (2a)

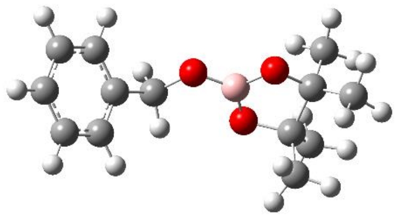

Sum of electronic and thermal Enthalpies= -757.013318

Sum of electronic and thermal Free Energies= -757.0727

O	-1.55397	0.66459	1.60306
C	-1.14436	1.81855	2.33272
H	-0.07348	1.76696	2.55881
H	-1.69598	1.78227	3.27638
C	-0.55752	-0.48988	-1.61926
C	0.27816	0.82885	-1.43774
B	-0.96923	0.43853	0.39816
O	-1.51330	-0.39936	-0.54118
O	0.20149	1.03676	-0.01240
C	-0.36989	2.04965	-2.09583
H	0.14120	2.94982	-1.74444
H	-0.29463	2.00490	-3.18627
H	-1.42464	2.13981	-1.81895
C	1.73871	0.72014	-1.84710
H	1.81993	0.46473	-2.90877
H	2.23693	1.68029	-1.68802
H	2.26354	-0.03712	-1.26208
C	0.26749	-1.75714	-1.39047

H	-0.41114	-2.61176	-1.32923
H	0.97064	-1.92958	-2.21061
H	0.82987	-1.70177	-0.45385
C	-1.31227	-0.58293	-2.93704
H	-0.61454	-0.55105	-3.78048
H	-1.86019	-1.52793	-2.98134
H	-2.03090	0.23144	-3.04329
C	-1.45382	3.09422	1.58070
C	-2.72963	3.29912	1.04766
C	-0.47171	4.06303	1.37889
C	-3.01498	4.45275	0.32318
H	-3.49355	2.53952	1.18778
C	-0.75702	5.22407	0.66273
H	0.53019	3.89777	1.76555
C	-2.02875	5.41977	0.12997
H	-4.00823	4.59889	-0.09030
H	0.01801	5.96905	0.51028
H	-2.25096	6.31966	-0.43523

TS-d

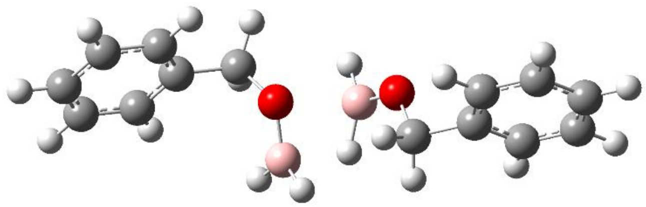

Sum of electronic and thermal Enthalpies= -744.022363

Sum of electronic and thermal Free Energies= -744.083866

B	0.16768	-1.71019	-1.99267
H	0.35702	-1.05464	-2.97415
C	1.03271	-2.25169	0.23364
H	0.10815	-2.81876	0.38641
H	-1.36571	-0.85172	-1.57652
B	-0.91514	-0.21574	-0.61889
O	-0.59019	1.08828	-0.78452

H	1.22187	-1.64640	1.12332
O	0.85350	-1.31651	-0.84392
C	-0.36721	1.65757	-2.05911
H	0.67961	1.48661	-2.34002
H	-0.99538	1.15772	-2.80899
H	-0.34331	-2.79445	-2.03656
H	-1.15371	-0.62286	0.47687

C	2.18561	-3.17636	-0.05400
C	1.96227	-4.51224	-0.38927
C	3.49738	-2.69458	-0.01300
C	3.03232	-5.35807	-0.67749
H	0.94525	-4.89335	-0.42755
C	4.56675	-3.53478	-0.30427
H	3.67607	-1.65376	0.24290
C	4.33537	-4.86992	-0.63622
H	2.84672	-6.39604	-0.93516
H	5.58155	-3.15100	-0.26958
H	5.16991	-5.52679	-0.86066

C	-0.66019	3.13933	-2.05304
C	-0.41860	3.87910	-3.21433
C	-1.16918	3.78608	-0.92883
C	-0.68534	5.24361	-3.25300
H	-0.01820	3.38310	-4.09592
C	-1.43565	5.15489	-0.96691
H	-1.35093	3.21394	-0.02591
C	-1.19636	5.88728	-2.12589
H	-0.49332	5.80579	-4.16178
H	-1.83135	5.64856	-0.08445
H	-1.40427	6.95236	-2.15306

References

(1) Lavergne, J. L.; Jayaraman, A.; Castro, L. C. M.; Rochette, É.; Fontaine, F.-G. *J. Am. Chem. Soc.* **2017**, *139*, 14714–14723.

(2) Rochette, É.; Bouchard, N.; Lavergne, J. L.; Matta, C. F.; Fontaine, F.-G. *Angew. Chem. Int. Ed.* **2016**, *55*, 12722–12726.

(3) Barman, M. K.; Baishya, A.; Nembenna, S. *Dalton Trans.* **2017**, *46*, 4152–4156.

(4) Barger, C. J.; Motta, A.; Weidner, V. L.; Lohr, T. L.; Marks, T. J. *ACS Catal.* **2019**, *9*, 9015–9024.

(5) Cao, X.; Wang, W.; Lu, K.; Yao, W.; Xue, F.; Ma, M. *Dalton Trans.* **2020**, *49*, 2776–2780.

(6) Ang, N. W. J.; Buettner, C. S.; Docherty, S.; Bismuto, A.; Carney, J. R.; Docherty, J. H.; Cowley, M. J.; Thomas, S. P. *Synth.* **2018**, *50*, 803–808.

(7) Austin, A.; Petersson, G. A.; Frisch, M. J.; Dobek, F. J.; Scalmani, G.; Throssell, K. *J. Chem. Theory Comput.* **2012**, *8*, 4989–5007.

(8) Chai, J. D.; Head-Gordon, M. *Phys. Chem. Chem. Phys.* **2008**, *10*, 6615–6620.

(9) Marenich, A. V.; Cramer, C. J.; Truhlar, D. G. *J. Chem. Theory Comput.* **2013**, *9*, 609–620.

(10) Grimme, S. *J. Comput. Chem.* **2006**, *32*, 1787–1799.
